# Design of platform trials with a change in the control treatment arm

**DOI:** 10.1093/biomtc/ujaf073

**Published:** 2025-06-20

**Authors:** Peter Greenstreet, Thomas Jaki, Alun Bedding, Pavel Mozgunov

**Affiliations:** Ottawa Methods Centre, Ottawa Hospital Research Institute, Ottawa, ON K1H 8L6, Canada; Department of Mathematics and Statistics, Lancaster University, Lancaster, LA1 4YF, United Kingdom; MRC Biostatistics Unit, University of Cambridge, Cambridge, CB2 0RS, United Kingdom; Department of Machine Learning and Data Science, University of Regensburg, Regensburg, 93053, Germany; Alun Bedding Coaching & Consulting Ltd, Bury St Edmunds, IP28 8PU, United Kingdom; MRC Biostatistics Unit, University of Cambridge, Cambridge, CB2 0RS, United Kingdom

**Keywords:** change in control, frequentist trials, multi-arm, multi-stage, platform trials

## Abstract

Platform trials are an efficient way of testing multiple treatments. We consider platform trials where, if a treatment is found to be superior to the control, it will become the new standard of care. The remaining treatments are then tested against this new control. In this setting, one can either keep the information on both the new standard of care and the other active treatments before the control is changed or discard this information when testing for benefit of the remaining treatments. We show analytically and numerically, retaining the information collected before the change in control can be detrimental to the power in a frequentist multi-arm multi-stage trial. Specifically, we consider the overall power, the probability that the active treatment with the greatest treatment effect is found during the trial, and the conditional power, the probability a given treatment is found superior against the current control. Also studied is the conditional type I error, the probability a given treatment is incorrectly found superior against the current control. We prove when retaining the information decreases both the overall and conditional power but also decreases the conditional type I error. A motivating example is then studied. Based on these observations, we discuss different aspects to consider when deciding whether to run a continuous platform trial or run an inherently new trial using the same trial infrastructure.

## 1 INTRODUCTION

Clinical trials take many years and are very costly to run (Mullard, 2018), which has led to multiple developments in methodology on how to efficiently design them (Pallmann et al., 2018). One of these developments has been the idea of platform trials in which multiple treatments are tested against a common control group (Wason and Jaki, 2012). Platform trials can be advantageous due to having a shared trial infrastructure and shared control groups (Burnett et al., 2024). The interest in these types of trials has increased since the beginning of the COVID-19 pandemic (Stallard et al., 2020), as platform trials can result in therapies being identified faster while reducing cost and time (Cohen et al., 2015).

One additional ability one may want from a platform trial’s design is to be able to change the control group to a beneficial new treatment found within the trial. A change of control has happened in multiple platform trials such as STAMPEDE (Sydes et al., 2012) and RECOVERY (Horby et al., 2021). In STAMPEDE, the control was changed as the standard of care changed during the trial, and in RECOVERY, there was no standard treatment, so it was changed once a treatment was shown to be superior. When changing the control group, one may think of using all the data collected to calculate the future test statistics. There is little work currently investigating whether keeping the data collected prior to the change of the control group treatment is the most efficient approach when allowing for a single change in control. In this paper, we will consider 2 settings: (1) We keep all the data from before the change in control and (2) we do not keep any of the data prior to the control changing. The 2 settings lead to the question: Would one be better off starting a new trial or continuing with the original trial when considering power assuming that the trial objective remains the same and the established trial infrastructure is retained.

This work will focus on frequentist multi-arm multi-stage trials (MAMS). Multi-arm trials allow for multiple treatments to be compared at once against a common control treatment. Multi-stage trials have interim analyses, which allow for ineffective treatments to be dropped for futility (or lack of benefit) earlier. As a result interim analyses can improve a trial’s operating characteristics (Pocock, 1977; Todd et al., 2001). They can also allow treatments to stop early if a superior treatment is found; however, in the case studied here, the first time this happens this superior treatment will become the new control.

We will focus our investigation on 2 types of power: (1) Conditional power of a treatment—the probability a given treatment can be found superior against the current control. (2) Overall power of the trial—the probability that the active treatment with the greatest treatment effect is found during the trial. The conditional power is considered as it is of interest under the assumption that one of the remaining treatments may be superior to the new control but has not yet been declared the winner. The overall power is also considered as a metric that the trial results in the best treatment being found in the trial overall. Additionally, in this manuscript, the conditional type I error is investigated, which is the probability that a given treatment is incorrectly found superior against the current control.

As seen in the RECOVERY trial (Horby et al., 2021), when a new standard of care was found within the trial, the objective of the trial remained testing for superiority for the remaining treatments. The motivating example studied in this paper, in Section 5, is based on the TAILoR trial (Pushpakom et al., 2015). However, the results in this manuscript are generalizable to other frequentist platform trials. The TAILoR trial was motivated by combination antiretroviral therapy increasing the risk of insulin resistance, obesity, and type 2 diabetes, predisposing factors for cardiovascular disease, in human immunodeficiency virus (HIV)-positive individuals. The TAILoR study was an adaptive trial studying 3 different doses of telmisartan compared to control to see which (if any) reduces insulin resistance in HIV-positive individuals on antiretrovirals. The inclusion/exclusion criteria detailed in Pushpakom et al. (2015) included requiring the participants to be HIV-positive adults; receiving antiretroviral therapy for at least 6 months; having no pre-existing diagnosis of type 1 or 2 diabetes; and not known to have consistently low blood pressure. The primary outcome measure was a reduction in HOMA-IR (Homeostatic Model Assessment of Insulin Resistance) (Singh and Saxena, 2010). Superiority comparisons continue to be of interest if one finds that one of the lower doses is superior to the control as one would only want to consider using a higher dose if it is superior to the current dose. The results of this work, however, apply to any frequentist platform trial where continued testing for superiority is of interest.

In Section 2, we introduce the notation, the null hypotheses of interest and discuss type I error. Section 3 studies the conditional power and conditional type I error for MAMS trials and gives theorems to when keeping the old data is guaranteed to be detrimental to conditional power but beneficial to conditional type I error. In Section 4, we give the formulation for the overall power along with its definition and give theorems to when keeping the old data is guaranteed to be detrimental for the overall power. Section 5 studies the motivating example. Finally, we discuss the considerations one needs to make when deciding whether to use the pre-change data or not.

## 2 NOTATION AND TYPE I ERROR CONTROL

Consider a clinical trial with up to $K$ experimental arms that will be tested against 1 common control arm. For simplicity, we have the primary outcome for each patient is independent and normally distributed with known variance $\sigma ^2$. In the Web Appendix D, the findings are generalized to the case where the test statistics use for the primary endpoint are asymptotically normal. Each active treatment is tested at $J$ analyses with $J-1$ interim analyses. Let $n_{k,j}$ denote the number of patients recruited to treatment $k$ by the end of stage $j$. For this paper, the focus will be on equal sample size and allocation ratio for each treatments and equally spaced interim analyses for all treatments, as this ensures equal pairwise error for each treatment without needing to have multiple boundary shapes (Greenstreet et al., 2024). Therefore, the number of patients recruited between interim analyses is equal for each treatment, so we define $n_j=n_{k,j}$ for all $k \in \lbrace 0, \ldots , K \rbrace$ and $j \in \lbrace 0, \ldots , J \rbrace$. The focus of this work will be on changing the control group once. We have $k^{\prime }_{j}$ denoting the current control treatment at stage $j$, where $k^{\prime } \in \lbrace 0, \ldots , K \rbrace$. At the beginning of the trial $k^{\prime }=0$, we let $n_{j^{\prime }}$ denote the number of patients recruited prior to treatment $k^{\prime }$ becoming the control at stage $j^{\prime }$ for all $j^{\prime } \in \lbrace 0, \ldots , J \rbrace$.

The null hypotheses of interest are $ H_{k^{\prime }1}: \mu _1 \le \mu _{k^{\prime }}, H_{k^{\prime }2}: \mu _2 \le \mu _{k^{\prime }}, {..}. , H_{k^{\prime }K}: \mu _K \le \mu _{k^{\prime }},$ where $\mu _1, \ldots , \mu _K$ are the mean responses on the $K$ experimental treatments and $\mu _{k^{\prime }}$ is the mean response of the current control group with $\mu _{0}$ being the mean response of the initial control. Each of the $K$ hypotheses is potentially tested at a series of analyses indexed by $j \in \lbrace j^{\prime }+1,\ldots ,J \rbrace$ where $j^{\prime }$ is the stage treatment $k^{\prime }$ becomes the control. At analysis $j$ for treatment $k$, to test $H_{k^{\prime }k}$, it is assumed that responses, $X_{k,i}$ and $X_{k^{\prime },i}$, from patients $i \in \lbrace 1,\ldots , n_{j}\rbrace $ on treatments $k$ and $k^{\prime }$, are observed, respectively. These hypotheses are tested at given analysis $j$ using the test statistic


\begin{align*}
Z_{k,k^{\prime },j}=& \frac{ \sum ^{n_{j}}_{i=1} X_{k,i} - \sum ^{n_{j}}_{i=1} X_{k^{\prime },i} }{\sigma \sqrt{2(n_{j})}}.
\end{align*}

If only the data post the change in the control is used, then the test statistics are


\begin{align*}
Z^\star _{k,k^{\prime },j,j^{\prime }}= \frac{ \sum ^{n_{j}}_{i=n_{j^{\prime }}+1} X_{k,i} - \sum ^{n_{j}}_{i=n_{j^{\prime }}+1} X_{k^{\prime },i} }{\sigma \sqrt{2(n_{j}-n_{j^{\prime }})}}.
\end{align*}

These test statistics are used to test $H_{k^{\prime }k}$, so are used for the decision-making. Upper and lower stopping boundaries, $U=(u_{1},\ldots ,u_{J})$ and $L=(l_{1},\ldots ,l_{J})$, are used as follows. If $ Z_{k,k^{\prime },j}> u_{j}$, then $H_{k^{\prime }k}$ is rejected and the conclusion that treatment $k$ is superior to the current control is made. If $ Z_{k,k^{\prime },j}< l_{j}$, then treatment $k$ is dropped from all subsequent stages of the trial. If the $Z$ statistics for all the treatments fall below their lower boundary, then the trial stops for futility. Treatment $k$ and control continues to its next stage if $l_{j} \le Z_{k,k^{\prime },j} \le u_{j}$. If the post change data is only used, then the same rules apply now replacing $Z_{k,k^{\prime },j}$ with $Z^\star _{k,k^{\prime },j,j^{\prime }}$. If multiple treatments exceed the upper boundary at the same time point, following Magirr et al. (2012), then the treatment with the largest test statistic becomes the new control.

These upper and lower stopping boundaries are group-sequential bounds which are pre-defined in order to control the original type I error control aimed for in the original trial. Therefore, for example, they could be aiming to control the pairwise error rate (Choodari-Oskooei et al., 2020), the family wise error rate (FWER) (Burnett et al., 2024; Magirr et al., 2012), or the false discovery rate (Robertson et al., 2022). Typically, when continuing to use the same boundaries as already pre-defined, there is no longer a guarantee that this will control the type I error of interest after the change. This is because the original bounds were not designed for this. In Section 3, we study the effect on conditional type I error, which is the probability of making a type I error for a given active treatment after the control has changed.

## 3 CONDITIONAL POWER AND CONDITIONAL TYPE I ERROR

The conditional power is the probability that a treatment is found to be superior to the new control treatment. The conditional power is considered as one should only continue the trial if they believe that one of the remaining treatments may be superior to the new control but has not yet been declared the winner, and the conditional power is the probability of finding this winner.

Definition 1: The conditional power for treatment $k^\star$, given $k^{\prime }$ becomes the new standard of care at stage $j^{\prime }$, is the probability that treatment $k^\star$ is found superior to treatment $k^{\prime }$ by the end of the trial, so by stage $J$.

The conditional power can be split into 3 events. Event 1 ($E^1_{k^\star ,k^{\prime },j^{\prime }}$) is the event that treatment $k^{\prime }$ becomes the control at stage $j^{\prime }$. Event 2 ($E^2_{k^\star ,k^{\prime },j^{\prime }}$) is that treatment $k^\star$ is still in the trial when treatment $k^{\prime }$ becomes the control. Event 3 ($E^3_{k^\star ,k^{\prime },j^{\prime }}$) is that none of the other $k$ treatments become the control. The detailed formulations for $E^1_{k^\star ,k^{\prime },j^{\prime }}$, $E^2_{k^\star ,k^{\prime },j^{\prime }}$, $E^3_{k^\star ,k^{\prime },j^{\prime }}$ are given in Web  Appendix A. The conditional power is, $ P(\text{reject } H_{k^{\prime }k^\star }| E^1_{k^\star ,k^{\prime },j^{\prime }} \cap E^2_{k^\star ,k^{\prime },j^{\prime }} \cap E^3_{k^\star ,k^{\prime },j^{\prime }}).$

In order to equate the conditional power, one can use the conditional probability definition to remove the need to calculate any highly truncated multivariate normal distributions. The conditional power is


\begin{align*}
\frac{P(E^1_{k^\star ,k^{\prime },j^{\prime }} \cap E^2_{k^\star ,k^{\prime },j^{\prime }} \cap E^3_{k^\star ,k^{\prime },j^{\prime }} \cap E^4_{k^\star ,k^{\prime },j^{\prime }})}{P(E^1_{k^\star ,k^{\prime },j^{\prime }} \cap E^2_{k^\star ,k^{\prime },j^{\prime }} \cap E^3_{k^\star ,k^{\prime },j^{\prime }})},
\end{align*}

where $E^4_{k^{\prime },k^\star ,j^{\prime }}$ is the event that we $\text{reject } H_{k^{\prime }k^\star }$ within the rest of the trial. The formulations for $E^1_{k^\star ,k^{\prime },j^{\prime }}$, $E^2_{k^\star ,k^{\prime },j^{\prime }}$, $E^3_{k^\star ,k^{\prime },j^{\prime }}$, and $E^4_{k^\star ,k^{\prime },j^{\prime }}$ are given in Web Appendix A. This can be calculated using multivariate normal distributions as discussed for the motivating example.

In the case of only considering the data post changing the control, the test statistics before the change are now independent of the test statistics post the change. Therefore, one only needs the event that we $\text{reject } H_{k^{\prime }k}$ within the rest of the trial. For the case where only the post change data is used, we define this as $E^{\star 4}_{k^\star ,k^{\prime },j^{\prime }}$. The formulations for $E^{\star 4}_{k^\star ,k^{\prime },j^{\prime }}$ is given in Web Appendix A. The conditional power in this case is $ P(E^{\star 4}_{k^\star ,k^{\prime },j^{\prime }}) .$ Once again this can be calculated using multivariate normal distributions as discussed for the motivating example.

It can be proven that, in many cases, there is never a benefit to retaining the information pre-change in control treatment when considering conditional power and using the predefined boundaries. The first theorem (Theorem 1) states that if there is only 1 stage left and the upper boundary is positive, then keeping the historic data is detrimental to the conditional power.

Theorem 1: If a treatment $k^{\prime }$ becomes the control group treatment at stage $J-1$ ($E^1_{k^\star ,k^{\prime },J-1} \cap E^3_{k^{\prime },k^{\prime },J-1}$) and $u_J \ge 0$, then the conditional power for treatment $k^\star$, when retaining the data before the control changed, is less than or equal to the conditional power for treatment $k^\star$ when not retaining the pre-change data.

The proof of Theorem 1 is given in Web Appendix B. Theorem 1 uses the fact that the active treatment of interest must have been no better than the new control group at the point of changing the control group. If this was not the case, then the active treatment of interest would be the new control. Therefore, by keeping the data before changing control, one is disadvantaging the active treatment as one retains the fact that the active treatment has so far been found worse than the new control treatment. This theorem can be further extended. First, in Theorem 2, which states that if there are multiple stages of the trial left and both the upper and lower boundaries are greater than or equal to 0, then retaining the post change data is detrimental to the conditional power. The second extension is Theorem 3 which states that if there are multiple stages of the trial left and the upper boundaries are positive and there is no lower boundaries, then retaining the post change data is detrimental to the conditional power.

Theorem 2: If a treatment $k^{\prime }$ becomes the new control group treatment at stage $j^{\prime }$ ($E^1_{k^\star ,k^{\prime },j^{\prime }} \cap E^3_{k^{\prime },k^{\prime },j^{\prime }}$) and $u_j \ge 0$ and $l_j \ge 0$ for all $j \in \lbrace (j^{\prime }+1), \ldots , J\rbrace$, then the conditional power for treatment $k^\star$ when retaining the data before the control changed is less than or equal to the conditional power for treatment $k^\star$ when not retaining the pre-change data.

Theorem 3: If a treatment $k^{\prime }$ becomes the new control group treatment at stage $j^{\prime }$ ($E^1_{k^\star ,k^{\prime },j^{\prime }} \cap E^3_{k^{\prime },k^{\prime },j^{\prime }}$) and $u_j \ge 0$ and there are no lower boundaries for all $j \in \lbrace (j^{\prime }+1), \ldots , J\rbrace$, then the conditional power for treatment $k^\star$, when retaining the data before the control changed, is less than or equal to the conditional power for treatment $k^\star$ when not retaining the pre-change data.

The proof for Theorems 2 and 3 is given in Web Appendix B. Furthermore, as shown in Web Appendix I, even if $l_j < 0$ for some $j \in \lbrace (j^{\prime }+1) \ldots J\rbrace$, then one will find that retaining the old information is likely detrimental for the conditional power. However, in Web Appendix J, it is shown that there are cases when $l_j < 0$ where keeping the old data can be beneficial for conditional power. It is worth noting that a lower conditional power does not necessarily imply generally worse properties of a design. For example, in the case of treatment $k$ being inferior to treatment $k^{\prime }$, there is an increased chance of the wrong decision being made if the data pre-change is not retained. We define this event as the conditional type I error for a treatment.

Definition 2: The conditional type I error for treatment $k^\star$, given $k^{\prime }$ becomes the new standard of care at stage $j^{\prime }$, is the probability that treatment $k^\star$ is found superior to treatment $k^{\prime }$ by the end of the trial, given that $\mu _{k^\star } \le \mu _{k^{\prime }}$.

The conditional type I error is therefore the probability that a given treatment is found superior to a new control when in fact it is not superior. The conditional type I error is calculated using the same equations as the conditional power when $\mu _{K^\star } \le \mu _{k^{\prime }}$, so Theorems 1, 2, and 3 also hold. Therefore, when these theorems are true, the conditional type I error is decreased when retaining the pre-change data. Section 5 shows how much both the conditional power and conditional type I error changes between retaining the data or not for the motivating example.

## 4 OVERALL POWER

The overall power is the probability that during the trial, the active treatment with the greatest positive treatment effect is either taken forward as the new control or is declared superior compared to a new control, if the control has already changed.

Definition 3: The overall power for the treatment $k^\star$ which has the greatest treatment effect, $\mu _{k^\star } \ge \mu _k \forall k \in \lbrace 1, \ldots , K\rbrace$ equals the probability treatment $k^\star$ is found to be the new control or subsequently treatment $k^\star$ is found to be superior to a new control $k^{\prime }$, where $k^{\prime }\in \lbrace 1,\ldots ,k^\star -1,k^\star +1,\ldots , K \rbrace$.

Therefore, overall power for the treatment $k^\star$ which has the greatest treatment effect, $\mu _{k^\star } \ge \mu _k \forall k \in \lbrace 1,\ldots , K \rbrace $ can be calculated as


\begin{align*}
&&P\bigg {\lbrace } \bigcup ^{J}_{j^\star =1}(E^1_{k^\star ,k^\star ,j^\star } \cap E^3_{k^\star ,k^\star ,j^\star }) \cup \bigcup _{k^{\prime }\in \lbrace 1,\ldots ,K \rbrace /k^\star } \\
&&\bigcup ^{J}_{j^{\prime }=1}(E^1_{k^{\prime },k^\star ,j^{\prime }} \cap E^2_{k^{\prime },k^\star ,j^{\prime }} \cap E^3_{k^{\prime },k^\star ,j^{\prime }} \cap E^4_{k^{\prime },k^\star ,j^{\prime }}) \bigg {\rbrace }.
\end{align*}

Due to the multiple disjoint sets within Definition 3, the overall power can be split into multiple, easy to compute, parts. The first of these is the probability that at each interim $j^\star$, treatment $k^\star$ becomes the control ($\Xi _{k^\star ,j^\star }$) and this equals


(1) \begin{eqnarray*}
\Xi _{k^\star ,j^\star }=P(E^1_{k^\star ,k^\star ,j^\star } \cap E^3_{k^\star ,k^\star ,j^\star }).
\end{eqnarray*}

The probability that another treatment becomes the new control and then this treatment is found to be better than the new control ($\Omega _{k^\star ,k^{\prime },j^{\prime }}$) can be split into every possible $k^{\prime }$ and $j^{\prime }$


(2) \begin{eqnarray*}
\Omega _{k^\star ,k^{\prime },j^{\prime }}= P(E^1_{k^{\prime },k^\star ,j^{\prime }} \cap E^2_{k^{\prime },k^\star ,j^{\prime }} \cap E^3_{k^{\prime },k^\star ,j^{\prime }} \cap E^4_{k^{\prime },k^\star ,j^{\prime }}).
\end{eqnarray*}

Combining Equations (1) and (2), the overall power is $\sum ^{J}_{j^\star =1} \Xi _{k^\star ,j^\star } + \sum _{k^{\prime }\in \lbrace 1,\ldots ,K \rbrace /k^\star } \sum ^{J}_{j^{\prime }=1}\Omega _{k^{\prime },k^\star ,j^{\prime }}.$ When we consider only using the data post change in control, the probability that another treatment becomes the new control and then this treatment is found to be better than the new control ($\Omega ^\star _{k^\star ,k^{\prime },j^{\prime }}$) becomes, $\Omega ^\star _{k^\star ,k^{\prime },j^{\prime }}= P(E^1_{k^\star ,k^{\prime },j^{\prime }} \cap E^2_{k^\star ,k^{\prime },j^{\prime }} \cap E^3_{k^\star ,k^{\prime },j^{\prime }})P(E^{\star 4}_{k^\star ,k^{\prime },j^{\prime }}) .$ This is due to the independence of event 4 with the rest of the events. Therefore the overall power is $\sum ^{J}_{j^\star =1} \Xi _{k^\star ,j^\star } + \sum _{k^{\prime }\in \lbrace 1,\ldots ,K \rbrace /k^\star }\sum ^{J}_{j^{\prime }=1}\Omega ^\star _{k^\star ,k^{\prime },j^{\prime }}.$ One can prove when it is guaranteed that there is no benefit to retaining the information pre-change in control treatment when considering overall power. Based on Theorems 2 and 3 for the conditional power, one can prove similar results for the overall power.

Theorem 4: If $u_j \ge 0$ and $l_j \ge 0$ for all $j \in \lbrace 1, \ldots , J\rbrace$, then the overall power when retaining the data before the control changed is less than or equal to the overall power when not retaining the pre-change data.

Theorem 5: If $u_j \ge 0$ and there are no lower boundaries for all $j \in \lbrace 1, \ldots , J\rbrace$, then the overall power when retaining the data before the control changed is less than or equal to the overall power when not retaining the pre-change data.

The proof for Theorems 4 and 5 is given in Web Appendix C. Furthermore, as is shown in Web Appendix I, even if $l_j < 0$ for any $j \in \lbrace 1, \ldots , J\rbrace$, then there are cases that retaining information pre-change in the control group reduces overall power. This is shown in the example in Web Appendix I as the difference in conditional power between keeping and discarding the pre-change data is negative, therefore, so will the overall power.

## 5 MOTIVATING TRIAL EXAMPLE

We consider the motivating trial of TAILoR (Pushpakom et al., 2015). The TAILoR trial was a 4 arm trial which studied the effect of different doses of a treatment on HIV. The study had 1 interim analysis. We are going to use the operating characteristics from this study to see the effects on overall and conditional power if the control was changed mid trial if a treatment was found superior. In the original design, the FWER (Pushpakom et al., 2015) was controlled at 5% one sided for a normal continuous endpoint and there was a planned 90% power. The trial was planned to have equal allocation across stages. In addition, the clinically relevant effect, $\theta _1$, was set to $ 0.545$ and uninteresting effect, $\theta _0$, set to 0.178 with variance $\sigma ^2=1$.

Triangular stopping boundaries will be used (Whitehead, 1997) as recommended in Wason and Jaki (2012). The stopping boundaries will be calculated using the approach given in Magirr et al. (2012) to control FWER for the design before the change in control. The calculations of the power will be done using Greenstreet et al. (2025) in order to control the pairwise power for each treatment. This is chosen as it is similar to that used in the original trial but is designed for trials that continue after a treatment is taken forward. The calculations were carried out using R (R Core Team, 2021) with the method given here having the multivariate normal probabilities being calculated using the package mvtnorm (Genz et al., 2021); the upper and lower boundaries found using MAMS (Jaki et al., 2019) and the code was parallelized using packages doParallel (Daniel et al., 2022a) and foreach (Daniel et al., 2022b). The code is available in the Supplementary Materials.

### 5.1 Boundaries and sample size

Using the approach by Magirr et al. (2012), the triangular upper and lower stopping boundaries are found to be


\begin{align*}
U={\begin{pmatrix}u_{1} & u_{2}\\
\end{pmatrix}}= {\begin{pmatrix}2.358 & 2.223\\
\end{pmatrix}}, \,\, \,\, \,\, L={\begin{pmatrix}l_{1} & l_{2}\\
\end{pmatrix}}= {\begin{pmatrix}0.786 & 2.223\\
\end{pmatrix}}.
\end{align*}

Using Greenstreet et al. (2025), the maximum sample size is 344 based on 43 patients per arm per stage to ensure pairwise power of 90%.

### 5.2 Conditional power and conditional type I error

There is only 1 place in the trial where either the conditional power or conditional type I error is not 0 as there is only 1 interim analysis in the study. This happens when a treatment becomes the new control at the first stage. The conditional power for treatment $k$ when treatment $k^{\prime }$ becomes the new control at stage 1 is


(3) \begin{eqnarray*}
\frac{P(E^1_{k,k^{\prime },1} \cap E^2_{k,k^{\prime },1} \cap E^3_{k,k^{\prime },1} \cap E^4_{k,k^{\prime },1})}{P(E^1_{k,k^{\prime },1} \cap E^2_{k,k^{\prime },1} \cap E^3_{k,k^{\prime },1})}.
\end{eqnarray*}

When we only retain the new information, the conditional power is $P(E^{\star 4}_{k^\star ,k^{\prime },1}).$ In Web Appendix E, the formulations used to calculate $P(E^{\star 4}_{k^\star ,k^{\prime },1})$ and Equation (3) are given. Both equations are also used to calculate the conditional type I error.

### 5.3 Overall power

The overall power for treatment $k^\star$ when the old information is retained is $ \sum ^{2}_{j^\star =1} \Xi _{k^\star ,j^\star } + \sum _{k^{\prime }\in \lbrace 1,2,3\rbrace /k^\star }\Omega _{k^\star ,k^{\prime },1}.$ When only new data is used, the overall power is $ \sum ^{2}_{j^\star =1} \Xi _{k^\star ,j^\star } +\sum _{k^{\prime }\in \lbrace 1,2,3\rbrace /k^\star } \Omega ^\star _{k^\star ,k^{\prime },1}.$ The formulation for $\Omega _{k^\star ,k^{\prime },1}$, $\Omega ^\star _{k^\star ,k^{\prime },1} \Xi _{k,1}$, and $\Xi _{k,2}$ are given in Web Appendix F.

### 5.4 Results

**FIGURE 1:**
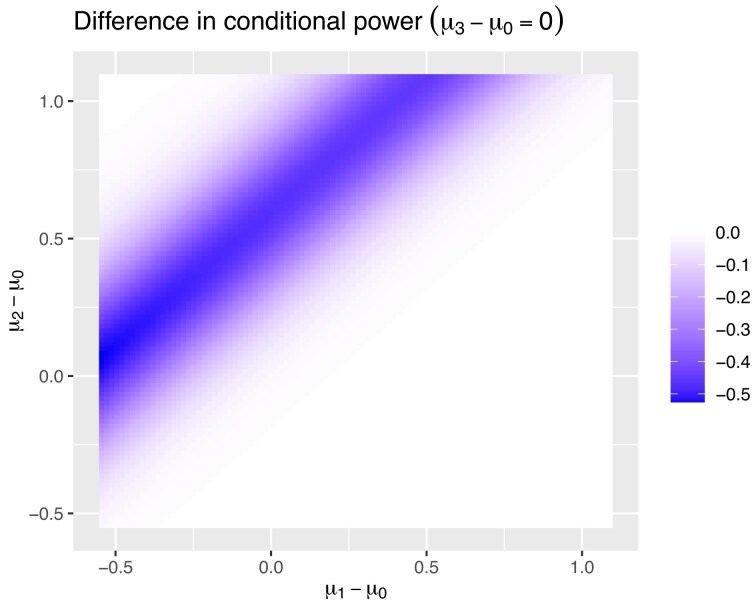
The difference in conditional power between keeping the data pre-change and not, for treatment 2 given that treatment 1 has gone forward at the first stage.

In Figure 1, the difference between conditional power when retaining all the old data and not retaining the data can be seen. The conditional power for treatment 2, when treatment 1 is the new control after the first stage, is studied. The *y*-axis gives the treatment effect of treatment 2 compared to the original control and the *x*-axis gives effect of treatment 1 compared to the original control. The color as given on the scale, to the right of the figure, defines the difference in conditional power between retaining the information pre the change and not. The effect of different values of $\mu _3$ is very small, as it has very little effect on the probability that treatment 2 is found superior to treatment 1 in the final stage or vice versa. Therefore, we will focus on the results for when $\mu _3-\mu _0=0$. However, in Web Appendix G, the effect of $\mu _3-\mu _0$ having both a negative and positive uninteresting treatment effect are shown.

The difference between conditional power when retaining all the old data and not retaining the data can be seen in Figure 1 when $\mu _3-\mu _0=0$. As can be seen in Figure 1, when $\mu _2-\mu _1$ is around 0.5, then the loss in conditional power is maximized. This can be greater than 50%. However, as this difference becomes a lot more extreme, the loss becomes close to 0. As at this point, either approach has almost an 100% chance of finding treatment 2 superior to treatment 1. In Web Appendix H, we study the effect on conditional power of different possible values of $Z_{(1,0),1}$ and $Z_{(2,0),1}$ for one of these points, $\mu _1=-0.25$ and $\mu _2= 0.75$. Here, we can ignore the value of $Z_{(3,0),1}$ as this does not influence the probabilities as shown in the proof to Theorem 1 in Web Appendix B. It is shown here that even in this case where there is on average very little benefit in only retaining the new information, there are potential values of $Z_{(1,0),1}$ and $Z_{(2,0),1}$ where there is large benefit in only using the new data. However, the probability of these $Z$ values happening is very small for the given $\mu _1$ and $\mu _2$.

**FIGURE 2:**
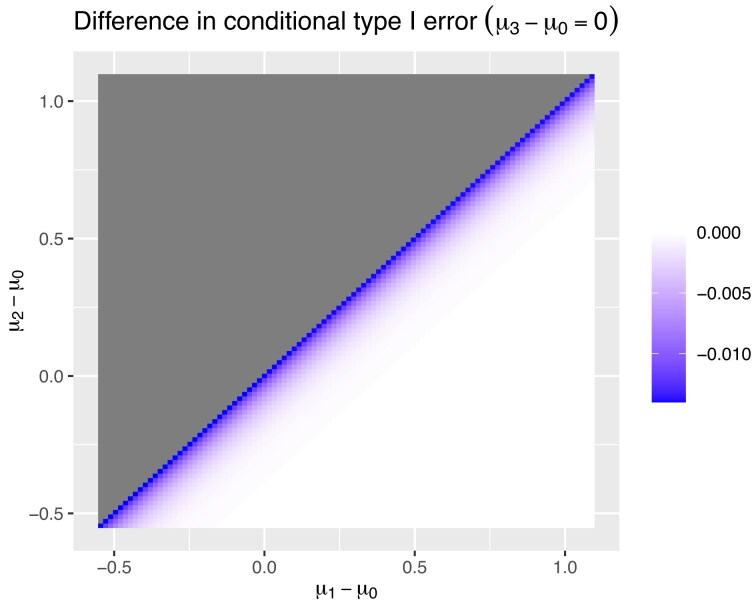
The difference in conditional type I error between keeping the data pre-change and not, for treatment 2 given that treatment 1 has gone forward at the first stage. The gray area defines the section where $\mu _2>\mu _1$ so no conditional type I error can be made.

Figure 2 gives the difference in conditional type I error when comparing keeping or disregarding the data pre-change in control. The top left of the figure is grayed out for all the values of $\mu _2>\mu _1$ as no conditional type I error would be made. The inflation shown in Figure 2 is smaller than the increase seen for the conditional power with the maximum inflation in conditional type I error of 1.40% which happens when $\mu _2 = \mu _1$. This is because, for both approaches, the probability that treatment 2 is found superior to treatment 1 when in fact it is not, is small. However, this clearly demonstrates that disregarding the data pre-change can increase the conditional type I error.

**FIGURE 3:**
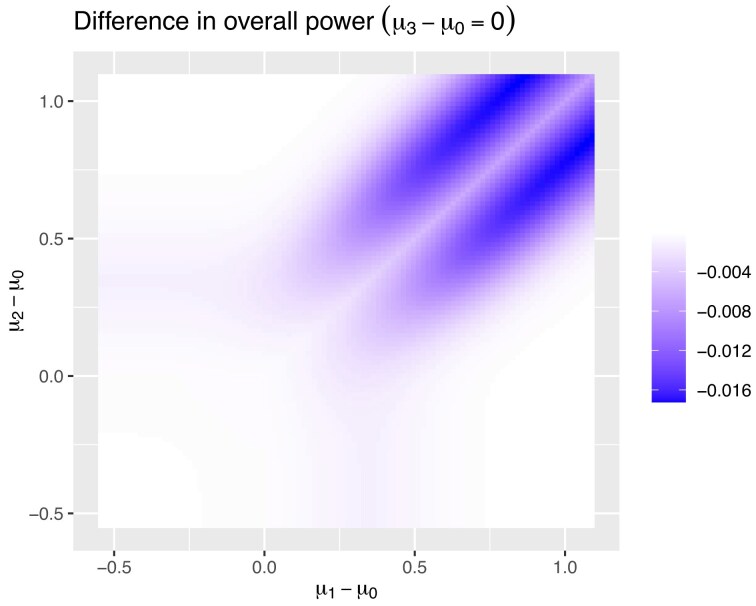
The difference in overall power between keeping the data pre-change and not.

In Figure 3, the difference between overall power when retaining all the old data and not retaining the data can be seen. As it was for conditional power, the effect of different values of $\mu _3$ is very small, so we will focus on the results for when $\mu _3-\mu _0=0$.

**FIGURE 4:**
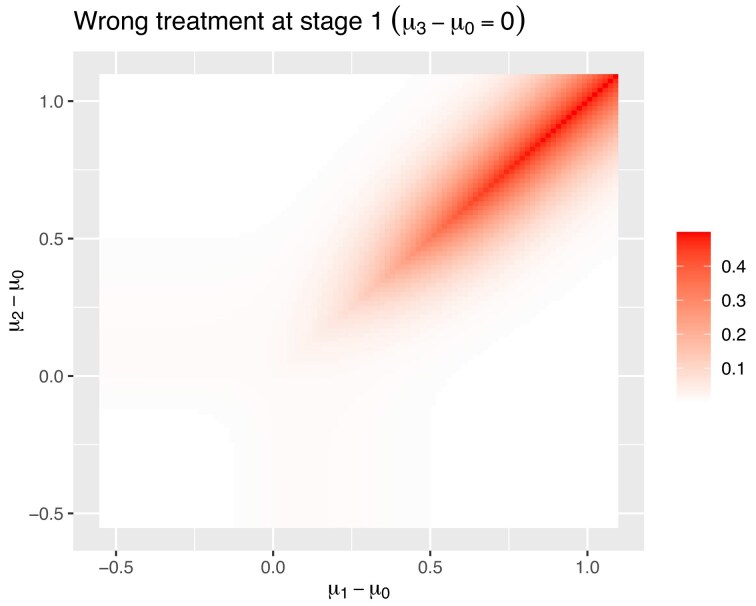
The probability of the treatment which does not have the greatest treatment effect becoming the control at the first stage.

The maximum difference in overall power is 1.7%. When calculating the overall power, most of the time, the correct treatment will be taken forward compared to the original control instead of one of the other treatments, so there is not a huge difference between retaining the pre-change data or not. This effect can be seen in Figure 4. This figure gives the probability of the treatment which does not have the greatest treatment effect becoming the control at the first stage.

## 6 DISCUSSION

In this paper, we have studied the effect of keeping or discarding the data post a change in control in a specific type of platform trial. The platform trial type of focus has been frequentist MAMS studies where all the arms begin at once. This work has shown that, for this trial design, one is likely to be better off not retaining the data, when changing the control treatment, with respect to overall and conditional power. The reason for this result is that the active treatment of interest must have been found no better than the new control group at the time of changing the control. If this was not the case, then the active treatment of interest would be the new control. By retaining the data from before the change of control, one is disadvantaging the active treatment as it has so far been found worse then the new control treatment. It would likely be beneficial to start a new trial instead, under the assumption that one is able to use the established infrastructure. There are likely to be other benefits in starting a new trial, including being able to adjust: the research question, the treatments of interest, the target population, and the treatment doses. However, a new trial may involve more administrative and logistical work compared to continuing the trial, even if one can keep the same established infrastructure. Additionally, starting a new trial can increase the conditional type I error compared to retaining all the data, therefore, increasing the chance an inferior treatment being incorrectly declared superior. Other performance metrics of a trial will also be effected by the choice to retain or not that are not considered here, which is an area for further research. It is worth noting that one maybe required to start a new trial instead of continuing the platform trial, in which any further treatments are tested against the new standard of care and the original standard of care, which is an area for further research.

This work has focused on the situation when all treatments start recruitment at the same time; however, one may be interested in the setting of adding additional arms (Burnett et al., 2024; Greenstreet et al., 2024, 2025). The methodology section in this paper is extended in Web Appendix L to the setting of finding the overall and conditional power when additional arms are added in a pre-planned manner. Concurrent data means only participants recruited to the current control arm at the same time as the active arm of interest are used in the comparisons. In the setting discussed in Web Appendix L, there is now unequal information per treatment when changing the control, therefore it is not guaranteed that retaining the information pre-change will be detrimental. One should calculate the conditional and overall power for there given example to establish which approach is favorable. This is shown in 2 examples in Web Appendix L. In the first case, there is no benefit in keeping the data, but for the second case, there can be benefit in keeping the data, with respect to the power of the study.

In this paper, it has been assumed that the primary outcome on each patient is independent and normally distributed with known variance $\sigma ^2$. However, this work can be applied more generally to multiple different endpoints (Stallard and Todd, 2003; Jaki and Magirr, 2013) assuming asymptotic normality of the test statistics. In the Web Appendix D, the methodology for this is presented along with the additional assumptions required for the theorems in this work, when generalizing the results.

The findings of this work should also be extended to alternative platform trial designs, including using a Bayesian framework, using other adaptive features, and using other modes of pooling information between pre- and post-change in control, or changing to non-inferiority boundaries post the change in control. We believe that the results presented here will generalize to several other settings, as the active treatments will often still start with a disadvantage over the new control if the data is retained; however, further work is needed to study this.

Throughout this work, we considered the case of the original pre-defined stopping rules and sample size being retained. This is a realistic setting as the stopping rules where deemed the best for the objectives of the trial at the beginning of the study and hence continue being used in the light of unchanged objectives (ie, showing improvement over standard of care). Additionally, funding limits may restrict the ability to change the sample size of a study. It is, however, worth noting that the results presented in Sections 3 and 4 still hold if one uses different boundary shapes and sample sizes post changing the control as long as these are the same regardless of whether the data pre-change is retained or not.

Furthermore, in this work, we have looked at an ideal example where the trial has equal allocation as planned. However, in reality, the probability of having equal allocation is very slim depending on the treatment allocation method. Therefore, we have also considered the effect of using simple random allocation. This therefore means criteria of Theorem 2 or Theorem 3 are no longer met. We have investigated this for 3 cases. We studied the number of times out of 100 000 000 simulations that keeping the data has resulted in the treatment of interest being taken forward when this would not have been the case using only the new data. This probability is still very small (0.0006% in the example studied) relative to the probability that discarding the old data has resulted in the treatment of interest being taken forward when this would not have been the case using all the data. This can be seen in Web Appendix K.

Overall, this paper has highlighted the importance of considering what to do if you change control during a frequentist MAMS trial to one which has been found superior to the control. Therefore, one should consider if they should continue the current trial or stop and start a new trial with the new control using the same trial infrastructure.

## Supplementary Material

ujaf073_Supplemental_Files Web Appendices, data and code referenced in Sections 2, 3, 4, 5, 6 are available with this paper at the Biometrics website on Oxford Academic.

## Data Availability

The simulated data that support the findings in this paper are available from the code provided in the Supplementary Materials.
