# Supplementary material for: Design of platform trials with a change in the control treatment arm
Source: Biometrics. 2025 Jun 20;81(2):ujaf073. doi: 10.1093/biomtc/ujaf073 (PMC12204708; doi:10.1093/biomtc/ujaf073)
Supplement: ujaf073_Supplemental_Files — Web Appendices, data and code referenced in Sections 2, 3, 4, 5, 6 are available with this paper at the Biometrics website on Oxford Academic. [file ujaf073_supplemental_files.zip › Supplementary Materials for Design of platform trials with a change in the control treatment arm by Peter Greenstreet, et al.pdf]

Supplementary Materials for Design of platform trials with a change in the control treatment arm by Peter Greenstreet, Thomas Jaki, Alun Bedding, Pavel Mozgunov

Web Appendix A Formulation of the events for calculating conditional type I error, conditional power and overall power

The event $E^1_{k^\star,k',j'}$ which is the event that treatment k' becomes the control at stage j' equals,

$$E^1_{k^\star,k',j'} = \bigcap_{i=1}^{j'-1}(l_i \leq Z_{k',0,i} \leq u_i) \cap Z_{k',0,j'} \geq u_{j'}.$$

The event $E^2_{k^\star,k',j'}$ which is that treatment $k^\star$ is still in the trial when treatment k' becomes the control equals,

$$E^2_{k^\star,k',j'} = \bigcap_{i=1}^{j'-1}(l_i \leq Z_{k',0,i} \leq u_i) \cap \quad (l_{j'} \leq Z_{k^\star,0,j'}) \cap [(Z_{k^\star,0,j'} \leq u_{j'}) \cup (Z_{k^\star,k,j'} \leq 0)]$$

The event $E^3_{k^\star,k',j'}$ which is that none of the other k treatments become the control is

$$E^3_{k^\star,k',j'} = \bigcap_{k\in(1\ldots K)/k^\star,k'} \left\{ \bigcap_{i=1}^{j'-1}(l_i \leq Z_{k,0,i} \leq u_i) \cap [(Z_{k,0,j'} \leq u_{j'}) \cup (Z_{k,k',j'} < 0)] \right\}.$$

The event $E^4_{k',k^\star,j'}$ which is the event that we reject $H_{k'k}$ within the rest of the trial equals,

$$E^4_{k^\star,k',j'} = \bigcup_{i=j'+1}^{J} \bigcap_{i^\star=j'+1}^{i-1} (l_{i^\star} \leq Z_{k^\star,k',i^\star} \leq u_{i^\star}) \cap (u_i < Z_{k^\star,k',i}).$$

The event $E^{\star 4}_{k^\star,k',j'}$ which is the event that we reject $H_{k'k}$ within the rest of the trial when not retaining the information post the change in control treatment equals,

$$E^{\star 4}_{k^\star,k',j'} = \bigcup_{i=j'+1}^{J} \bigcap_{i^\star=j'+1}^{i-1} (l_{i^\star} \leq Z^\star_{k^\star,k',i^\star,j'} \leq u_{i^\star}) \cap (u_i < Z^\star_{k^\star,k',i,j'}).$$

Web Appendix B Proof of Theorem 1, Theorem 2 and Theorem 3

The proof of Theorem 1 is:

Proof. Define $\widehat{Z}_{k,k',j'}$, where $\widehat{Z}_{k,k',j'}$ equals $Z_{k,k',j}$ at $n_{j'}$, so:

$$\widehat{Z}_{k,k',j'} = \frac{\sum_{i=1}^{n_{j'}} X_{k,i} - \sum_{i=1}^{n_{j'}} X_{k',i}}{\sigma\sqrt{2(n_{j'})}}.$$

Therefore,

$$Z_{k,k',j} = \frac{\widehat{Z}_{k,k',j'}\sqrt{n_{j'}} + Z^{\star}_{k,k',j,j'}\sqrt{n_j - n_{j'}}}{\sqrt{n_j}}.$$

The same boundaries U and L, as predefined for the trial, are used so if the old data is kept one can rearrange $Z_{k,k',j} > u_j$ to be:

$$Z^{\star}_{k,k',j,j'} > \frac{u_j\sqrt{n_j} - \widehat{Z}_{k,k',j'}\sqrt{n_{j'}}}{\sqrt{n_j - n_{j'}}},$$

compared to $Z^{\star}_{k,k',j,j'} > u_j$ for only new data. There is only increased chance of going above u_j when keeping the historic data if:

$$\widehat{Z}_{k,k',j'} > \frac{u_j(\sqrt{n_j} - \sqrt{n_j - n_{j'}})}{\sqrt{n_{j'}}}. \qquad \text{(Web Appendix B.1)}$$

For an increased chance of rejecting the null hypothesis $H_{k,k'}$ at the next stage if pre change data is kept compared to discarding it one requires $\widehat{Z}_{k,k',j'}$ to be positive if u_j is positive. Using Equation (Web Appendix B.1) if all treatments are added at the same point it is worth keeping the historic data if:

$$\widehat{Z}_{k,k',j'} > \frac{u_j(\sqrt{n_j} - \sqrt{n_j - n_{j'}})}{\sqrt{n_{j'}}} \geq 0.$$

However $\widehat{Z}_{k,k',j'} < 0$ as treatment k' is the new control not treatment $k^{\star}$. □

The proof of Theorem 2 is:

Proof. Define the following:

$$B_{k^{\star},j}(\delta_{1,j}, \delta_{2,j}) = \{(\delta_{2,j}l_j + \delta_{1,j}) < Z^{\star}_{k^{\star},k',j} < (\delta_{2,j}u_j + \delta_{1,j})\}$$
$$C_{k^{\star},j}(\delta_{1,j}, \delta_{2,j}) = \{(\delta_{2,j}u_j + \delta_{1,j}) < Z^{\star}_{k^{\star},k',j}\}.$$

From Definition 1 the conditional power equals:

$$R(\delta_{1,j}, \delta_{2,j}) = \bigcup_{j=j'+1}^{J} \left[\bigcap_{i=j'+1}^{j-1} \{B_{k^{\star},i}(\delta_{1,i}, \delta_{2,i})\} \cap C_{k^{\star},j}(\delta_{1,j}, \delta_{2,j}) \right].$$

When no data is taken $\delta_{1,j} = 0$ and $\delta_{2,j} = 1$. However when old data is taken forward $\delta_{1,j} = \frac{-\widehat{Z}_{k,k',j'}\sqrt{n_{k',j'}}}{\sqrt{n_{k,j}-n_{k,j'}}}$ and $\delta_{2,j} = \frac{\sqrt{n_{k,j}}}{\sqrt{n_{k,j}-n_{k,j'}}}$. Therefore when old data is retained $\delta_{1,j} \geq 0$ and $\delta_{2,j} \geq 1$ as $\widehat{Z}_{k,k',j'} < 0$.

Then under the assumption $u_j \geq 0$ and $l_j \geq 0$ for all $j \in \{(j'+1), \ldots, J\}$. For any $\epsilon_{1,j} \geq 0$ and $\epsilon_{2,j} \geq 0$ let

$$w = (Z^{\star}_{k^{\star},k',j'+1}, \ldots, Z^{\star}_{k^{\star},k',J}) \in \bigcup_{j=j'+1}^{J} \Bigg[\bigcap_{i=j'+1}^{j-1} \{B_{k^{\star},i}(\delta_{1,i}+\epsilon_{1,i}, \delta_{2,i}+\epsilon_{2,i})\} \cap C_{k^{\star},j}(\delta_{1,j}+\epsilon_{1,j}, \delta_{2,j}+\epsilon_{2,j}) \Bigg],$$

for some $q \in \{j'+1, \ldots, J\}$ for which $Z^{\star}_{k^{\star},k',q} \in C_{k^{\star},q}(\delta_{1,q}+\epsilon_{1,q}, \delta_{2,q}+\epsilon_{2,q})$ and $Z^{\star}_{k^{\star},k',h} \in B_{k^{\star},h}(\delta_{1,h}+\epsilon_{1,h}, \delta_{2,h}+\epsilon_{2,h})$ for $h = j'+1, \ldots q-1$. $Z^{\star}_{k^{\star},k',q} \in C_{k^{\star},q}(\delta_{1,q}+\epsilon_{1,q}, \delta_{2,q}+\epsilon_{2,q})$ implies that $Z^{\star}_{k^{\star},k',q} \in C_{k^{\star},q}(\delta_{1,q}, \delta_{2,q})$. Furthermore $Z^{\star}_{k^{\star},k',q} \in B_{k^{\star},q}(\delta_{1,q}+\epsilon_{1,q}, \delta_{2,q}+\epsilon_{2,q})$ implies that $Z^{\star}_{k^{\star},k',q} \in B_{k^{\star},q}(\delta_{1,q}, \delta_{2,q}) \cup C_{k^{\star},q}(\delta_{1,q}, \delta_{2,q})$ for some $h = j'+1, \ldots q-1$. Therefore,

$$w = (Z^{\star}_{k^{\star},k',j'+1}, \ldots, Z^{\star}_{k^{\star},k',J}) \in \bigcup_{j=j'+1}^{J} \Bigg[\bigcap_{i=j'+1}^{j-1} \{B_{k^{\star},i}(\delta_{1,i}, \delta_{2,i})\} \cap C_{k^{\star},j}(\delta_{1,j}, \delta_{2,j}) \Bigg].$$

As a result $P\{R(0,1)\} \geq P\{R(\frac{-\widehat{Z}_{k,k',j'}\sqrt{n_{k',j'}}}{\sqrt{n_{k,j}-n_{k,j'}}}, \frac{\sqrt{n_{k,j}}}{\sqrt{n_{k,j}-n_{k,j'}}})\}$. □

The proof of Theorem 3 is:

Proof. We define the following:

$$\begin{aligned} B_{k^{\star},j}(\delta_{1,j}, \delta_{2,j}) &= \{-\infty < Z^{\star}_{k^{\star},k',j} < (\delta_{2,j}u_j + \delta_{1,j})\}, \\ C_{k^{\star},j}(\delta_{1,j}, \delta_{2,j}) &= \{(\delta_{2,j}u_j + \delta_{1,j}) < Z^{\star}_{k^{\star},k',j}\}. \end{aligned}$$

The conditional power equals is:

$$R(\delta_{1,j}, \delta_{2,j}) = \bigcup_{j=j'+1}^{J} \Bigg[\bigcap_{i=j'+1}^{j-1} \{B_{k^{\star},i}(\delta_{1,i}, \delta_{2,i})\} \cap C_{k^{\star},j}(\delta_{1,j}, \delta_{2,j}) \Bigg].$$

When no data is taken $\delta_{1,j} = 0$ and $\delta_{2,j} = 1$. However when old data is taken forward $\delta_{1,j} = \frac{-\widehat{Z}_{k,k',j'}\sqrt{n_{k',j'}}}{\sqrt{n_{k,j}-n_{k,j'}}}$ and $\delta_{2,j} = \frac{\sqrt{n_{k,j}}}{\sqrt{n_{k,j}-n_{k,j'}}}$. Therefore when old data is retained $\delta_{1,j} \geq 0$ and $\delta_{2,j} \geq 1$ as $\widehat{Z}_{k,k',j'} < 0$.

Then under the assumption $u_j \geq 0$ for all $j \in \{(j'+1), \ldots, J\}$. For any $\epsilon_{1,j} \geq 0$ and $\epsilon_{2,j} \geq 0$ let

$$w = (Z^{\star}_{k^{\star},k',j'+1}, \ldots, Z^{\star}_{k^{\star},k',J}) \in \bigcup_{j=j'+1}^{J} \Bigg[\bigcap_{i=j'+1}^{j-1} \{B_{k^{\star},i}(\delta_{1,i}+\epsilon_{1,i}, \delta_{2,i}+\epsilon_{2,i})\} \cap C_{k^{\star},j}(\delta_{1,j}+\epsilon_{1,j}, \delta_{2,j}+\epsilon_{2,j}) \Bigg].$$

For some $q \in \{j'+1,\dots,J\}$ for which $Z^\star_{k^\star,k',q} \in C_{k^\star,q}(\delta_{1,q}+\epsilon_{1,q},\delta_{2,q}+\epsilon_{2,q})$ and $Z^\star_{k^\star,k',h} \in B_{k^\star,h}(\delta_{1,h}+\epsilon_{1,h},\delta_{2,h}+\epsilon_{2,h})$ for $h=j'+1,\dots q-1$. $Z^\star_{k^\star,k',q} \in C_{k^\star,q}(\delta_{1,q}+\epsilon_{1,q},\delta_{2,q}+\epsilon_{2,q})$ implies that $Z^\star_{k^\star,k',q} \in C_{k^\star,q}(\delta_{1,q},\delta_{2,q})$. Furthermore $Z^\star_{k^\star,k',q} \in B_{k^\star,q}(\delta_{1,q}+\epsilon_{1,q},\delta_{2,q}+\epsilon_{2,q})$ implies that $Z^\star_{k^\star,k',q} \in B_{k^\star,q}(\delta_{1,q},\delta_{2,q}) \cup C_{k^\star,q}(\delta_{1,q},\delta_{2,q})$ for some $h=j'+1,\dots q-1$. Therefore,

$$w = (Z^\star_{k^\star,k',j'+1},\dots,Z^\star_{k^\star,k',J}) \in \bigcup_{j=j'+1}^{J}\left[\bigcap_{i=j'+1}^{j-1}\{B_{k^\star,i}(\delta_{1,i},\delta_{2,i})\} \cap C_{k^\star,j}(\delta_{1,j},\delta_{2,j})\right].$$

As a result $P\{R(0,1)\} \geq P\{R(\frac{-\widehat{Z}_{k,k',j'}\sqrt{n_{k',j'}}}{\sqrt{n_{k,j}-n_{k,j'}}},\frac{\sqrt{n_{k,j}}}{\sqrt{n_{k,j}-n_{k,j'}}})\}$. □

Web Appendix C Proof of Theorem 4 and Theorem 5

The proof of both Theorem 4 and Theorem 5 is:

Proof. Let the treatment with the greatest positive treatment effect be treatment $k^\star$. Then one can write the conditional power of treatment k if no pre change data is kept as:

$$\frac{P(E^1_{k^\star,k',j'} \cap E^2_{k^\star,k',j'} \cap E^3_{k^\star,k',j'})P(E^{\star 4}_{k^\star,k',j'})}{P(E^1_{k^\star,k',j'} \cap E^2_{k^\star,k',j'} \cap E^3_{k^\star,k',j'})}.$$

From Theorem 2 when $u_j \geq 0$ and $l_j \geq 0$ for all $j \in \{1,\dots,J\}$ is true we know for a given k' and j'

$$P(E^1_{k^\star,k',j'} \cap E^2_{k^\star,k',j'} \cap E^3_{k^\star,k',j'} \cap E^4_{k^\star,k',j'}) \leq P(E^1_{k^\star,k',j'} \cap E^2_{k^\star,k',j'} \cap E^3_{k^\star,k',j'})P(E^{\star 4}_{k^\star,k',j'}).$$

Additionally this is known for when $u_j \geq 0$ and there are no lower boundaries for all $j \in \{1,\dots,J\}$ from Theorem 3. This is true for every $k' \in \{1,\dots,K\}/k^\star$ and $j' \in 1,\dots,J$, so

$$\sum_{j^\star=1}^{J}\Xi_{k^\star,j^\star} + \sum_{k'\in\{1,\dots,K\}/k^\star}\sum_{j'=1}^{J}\Omega_{k^\star,k',j'} \leq \sum_{j^\star=1}^{J}\Xi_{k^\star,j^\star} + \sum_{k'\in\{1,\dots,K\}/k^\star}\sum_{j'=1}^{J}\Omega^\star_{k^\star,k',j'}.$$

□

Web Appendix D Generalised methodology when assuming asymptotic normality of test statistics

Similar to Stallard and Todd (2003) the null hypotheses of interest are

$$H_{k'1}:\theta_{1,k'} \leq 0, H_{k'2}:\theta_{2,k'} \leq 0, ..., H_{k'K}:\theta_{K,k'} \leq 0,$$

where $\theta_{1,k'},\dots,\theta_{K,k'}$ are the measure of the superiority of the experimental therapy compared to the control $k'=0,\dots,K$.

The test statistics are now:

$$Z_{k,k',j} = \frac{\overline{\psi}_{k,k',j}}{\sqrt{V_{(k,k'),j}}}.$$

where $\overline{\psi}_{k,k',j}$ is the treatment effect difference of the observed patients on treatments $k, k^\star$ upto the end of stage j and $V_{(k,k'),j}$ is the variance of the test statistic. It is assumed that $Z_{(k,k^\star),j}$ follows a normal distribution, $Z_{(k,k^\star),j} \sim (\overline{\psi}_{k,k',j}, V_{(k,k^\star),j})$. If only the data post the change in the control is used the test statistics are:

$$Z^\star_{k,k',j,j'} = \frac{\overline{\psi}^\star_{k,k',j,j'}}{\sqrt{V^\star_{(k,k'),j,j'}}}.$$

where $\overline{\psi}^\star_{k,k',j,j'}$ is the treatment effect difference of the observed patients on treatments $k, k^\star$ from the end of stage j' upto the end of stage j and $V^\star_{(k,k'),j,j'}$ is the variance of the test statistic. Additionally, similar to Stallard and Todd (2003), the treatment effect difference is set so $\overline{\psi}_{k,k',j} = \overline{\psi}_{k,k',j'} + \overline{\psi}^\star_{k,k',j,j'}$ given $j > j'$. Under this more general setting theorem 1 becomes:

Theorem Web Appendix D.1. *If a treatment k' becomes the control group treatment at stage $J-1$ ($E^1_{k^\star,k',J-1} \cap E^3_{k',k',J-1}$) and $u_J \geq 0$ then the conditional power for treatment $k^\star$ when retaining the data before the control changed is less than or equal to the conditional power for treatment $k^\star$ when not retaining the pre-change data assuming that $Z_{k,k',j'} \geq 0$ and $(\sqrt{V_{k,k',j}} - \sqrt{V^\star_{k,k',j,j'}}) > 0$.*

Proof. Define $\widehat{Z}_{k,k',j'}$, where $\widehat{Z}_{k,k',j'}$ equals $Z_{k,k',j}$ at stage j':

$$\widehat{Z}_{k,k',j'} = \frac{\overline{\psi}_{k,k',j'}}{\sqrt{V_{(k,k'),j'}}},$$

so $\widehat{Z}_{k,k',j'} = Z_{k,k',j'}$. Therefore,

$$Z_{k,k',j} = \frac{\widehat{Z}_{k,k',j'}\sqrt{V_{k,k',j'}} + Z^\star_{k,k',j,j'}\sqrt{V^\star_{k,k',j,j'}}}{\sqrt{V_{k,k',j}}}.$$

The same boundaries U and L, as predefined for the trial, are used so if the old data is kept one can rearrange $Z_{k,k',j} > u_j$ to be:

$$Z^\star_{k,k',j,j'} > \frac{u_j\sqrt{V_{k,k',j}} - \widehat{Z}_{k,k',j'}\sqrt{V_{k,k',j'}}}{\sqrt{V^\star_{k,k',j,j'}}},$$

compared to $Z^\star_{k,k',j,j'} > u_j$ for only new data. There is only increased chance of going above u_j when keeping the historic data if:

$$\widehat{Z}_{k,k',j'} > \frac{u_j(\sqrt{V_{k,k',j}} - \sqrt{V^\star_{k,k',j,j'}})}{\sqrt{V_{k,k',j'}}}. \quad \text{(Web Appendix D.1)}$$

For an increased chance of rejecting the null hypothesis $H_{k,k'}$ at the next stage if pre change data is kept compared to discarding it one requires $\widehat{Z}_{k,k',j'}$ to be positive if u_j is positive. Using Equation (Web Appendix D.1) if all treatments are added at the same point it is worth keeping the historic data if:

$$\widehat{Z}_{k,k',j'} > \frac{u_j(\sqrt{V_{k,k',j}} - \sqrt{V^{\star}_{k,k',j,j'}})}{\sqrt{V_{k,k',j'}}} \geq 0.$$

as $\sqrt{V_{k,k',j}} - \sqrt{V^{\star}_{k,k',j,j'}} > 0$. However as defined $\widehat{Z}_{k,k',j'} < 0$ so this is not true. □

Theorem 2-5 are the same as seen in Sections 3 and 4 of the main manuscript and can be proven in a similar way as seen in Web Appendix B and Web Appendix C, but now with the additional requirements that $Z_{k,k',j'} < 0$ and $\sqrt{V_{k,k',j}} - \sqrt{V^{\star}_{k,k',j,j'}} > 0$ is true for all $j, j' = 1, \ldots, J$. If the requirement of $Z_{k,k',j'} < 0$ is not met then this means that the best performing treatment so far was not taken forward to be the new control. Also if the requirement of that $\sqrt{V_{k,k',j}} - \sqrt{V^{\star}_{k,k',j,j'}} > 0$ is not met then this means there has been a lost in information by collecting more results. Therefore in almost all cases these requirements are met.

Web Appendix E Conditional power and conditional type I error formulations for the motivating trial example

The denominator of the conditional power and conditional type I error for a change after the first stage is:

$$P(E^1_{k^\star,k',1} \cap E^2_{k^\star,k',1} \cap E^3_{k^\star,k',1}) = \int_{u_1}^{\infty}\int_{l_1}^{\infty}\int_{-\infty}^{0}\int_{-\infty}^{0} \phi\left(\mathbf{z}, \mu^{\Omega_{k^\star,k',1}}_{[1,2,3,4]}, \Sigma^{\Omega_{k^\star,k',1}}_{[1,2,3,4]}\right)\mathbf{dz}. \quad \text{(Web Appendix E.1)}$$

where $\phi(\mathbf{z}, \mu, \Sigma)$ is the probability density function of a multi-variate normal distribution with mean μ and covariance matrix Σ. Due to each treatment getting the same number of patients per stage we define $n = n_1$ therefore the number of patients recruited at the second stage for a treatment which runs for both stages is $n + n = 2n$, so

$$\mu^{\Omega_{k^\star,k',1}} = \left(\frac{\sqrt{n}(\mu_{k'} - \mu_0)}{\sigma\sqrt{2}}, \frac{\sqrt{n}(\mu_{k^\star} - \mu_0)}{\sigma\sqrt{2}}, \frac{\sqrt{n}(\mu_{k^\star} - \mu_{k'})}{\sigma\sqrt{2}}, \frac{\sqrt{n}(\mu_{k} - \mu_{k'})}{\sigma\sqrt{2}}, \frac{\sqrt{n}(\mu_{k^\star} - \mu_{k'})}{\sigma}\right),$$

and

$$\Sigma^{\Omega_{k^\star,k',1}} = \begin{pmatrix} 1 & \frac{1}{2} & -\frac{1}{2} & -\frac{1}{2} & -\frac{1}{2}\sqrt{\frac{1}{2}} \\ \frac{1}{2} & 1 & \frac{1}{2} & 0 & \frac{1}{2}\sqrt{\frac{1}{2}} \\ -\frac{1}{2} & \frac{1}{2} & 1 & \frac{1}{2} & \sqrt{\frac{1}{2}} \\ -\frac{1}{2} & 0 & \frac{1}{2} & 1 & \frac{1}{2}\sqrt{\frac{1}{2}} \\ -\frac{1}{2}\sqrt{\frac{1}{2}} & \frac{1}{2}\sqrt{\frac{1}{2}} & \sqrt{\frac{1}{2}} & \frac{1}{2}\sqrt{\frac{1}{2}} & 1 \end{pmatrix},$$

with $[\cdot]$ defining which entries to take from the vector, and $[\cdot]$ also defines the rows and columns of the matrix, needed. The numerator of the conditional power for a change after the first stage is

$$P(E^1_{k^\star,k',1}\cap E^2_{k^\star,k',1}\cap E^3_{k^\star,k',1}\cap E^4_{k^\star,k',1}) = \int_{u_1}^{\infty}\int_{l_1}^{\infty}\int_{-\infty}^{0}\int_{-\infty}^{0}\int_{u_2}^{\infty}\phi\Big(\mathbf{z}, \mu^{\Omega_{k^\star,k',1}}, \Sigma^{\Omega_{k^\star,k',1}}\Big)\mathbf{dz}. \quad \text{(Web Appendix E.2)}$$

The conditional power for treatment $k^\star$ when treatment k' becomes the new control at stage 1 equals Equation (Web Appendix E.2) divided by Equation (Web Appendix E.1). When only retaining the new information the conditional power is

$$P(E^{\star 4}_{k^\star,k',1}) = \int_{u_2}^{\infty}\phi\Big\{\mathbf{z}, \Big(\frac{\sqrt{n}(\mu_{k^\star}-\mu_{k'})}{\sigma\sqrt{2}}\Big), 1\Big\}\mathbf{dz}. \quad \text{(Web Appendix E.3)}$$

Web Appendix F Overall power formulations for the motivating trial example

Due to all the arms starting at the same point $\Xi_{k,1}$ can be simplified to

$$\Xi_{k,1} = \int_{u_1}^{\infty}\int_{-\infty}^{0}\int_{-\infty}^{0}\phi\Big(\mathbf{z}, \mu^{\Xi_{k,1}}, \Sigma^{\Xi_{k,1}}\Big)\mathbf{dz},$$

where

$$\mu^{\Xi_{k,1}} = \Big(\frac{\sqrt{n}(\mu_{k^\star}-\mu_0)}{\sigma\sqrt{2}}, \frac{\sqrt{n}(\mu_{k_1}-\mu_{k^\star})}{\sigma\sqrt{2}}, \frac{\sqrt{n}(\mu_{k_2}-\mu_{k^\star})}{\sigma\sqrt{2}}\Big),$$

and

$$\Sigma^{\Xi_{k,1}} = \begin{pmatrix} 1 & -\frac{1}{2} & -\frac{1}{2} \\ -\frac{1}{2} & 1 & \frac{1}{2} \\ -\frac{1}{2} & \frac{1}{2} & 1 \end{pmatrix}.$$

The probability $k^\star$ becomes the control at the second stage is

$$\begin{aligned}\Xi_{k,2} = &\int_{l_1}^{u_1}\int_{u_2}^{\infty}\int_{-\infty}^{l_1}\int_{-\infty}^{l_1}\phi\Big(\mathbf{z}, \mu^{\Xi_{k,2}}_{[1,2,3,5]}, \Sigma^{\Xi_{k,2}}_{[1,2,3,5]}\Big)\mathbf{dz}+\\ &\int_{l_1}^{u_1}\int_{u_2}^{\infty}\int_{l_1}^{u_1}\int_{-\infty}^{0}\int_{-\infty}^{l_1}\phi\Big(\mathbf{z}, \mu^{\Xi_{k,2}}_{[1,2,3,4,5]}, \Sigma^{\Xi_{k,2}}_{[1,2,3,4,5]}\Big)\mathbf{dz}+\\ &\int_{l_1}^{u_1}\int_{u_2}^{\infty}\int_{-\infty}^{l_1}\int_{l_1}^{u_1}\int_{-\infty}^{0}\phi\Big(\mathbf{z}, \mu^{\Xi_{k,2}}_{[1,2,3,5,6]}, \Sigma^{\Xi_{k,2}}_{[1,2,3,5,6]}\Big)\mathbf{dz}+\\ &\int_{l_1}^{u_1}\int_{u_2}^{\infty}\int_{l_1}^{u_1}\int_{-\infty}^{0}\int_{l_1}^{u_1}\int_{-\infty}^{0}\phi\Big(\mathbf{z}, \mu^{\Xi_{k,2}}, \Sigma^{\Xi_{k,2}}\Big)\mathbf{dz}.\end{aligned}$$

where

$$\mu^{\Xi_{k,2}} = \Big(\frac{\sqrt{n}(\mu_{k^\star}-\mu_0)}{\sigma\sqrt{2}}, \frac{\sqrt{n}(\mu_{k^\star}-\mu_0)}{\sigma}, \frac{\sqrt{n}(\mu_{k_1}-\mu_0)}{\sigma\sqrt{2}}, \frac{\sqrt{n}(\mu_{k_1}-\mu_{k^\star})}{\sigma}, \frac{\sqrt{n}(\mu_{k_2}-\mu_0)}{\sigma\sqrt{2}}, \frac{\sqrt{n}(\mu_{k_2}-\mu_{k^\star})}{\sigma}\Big),$$

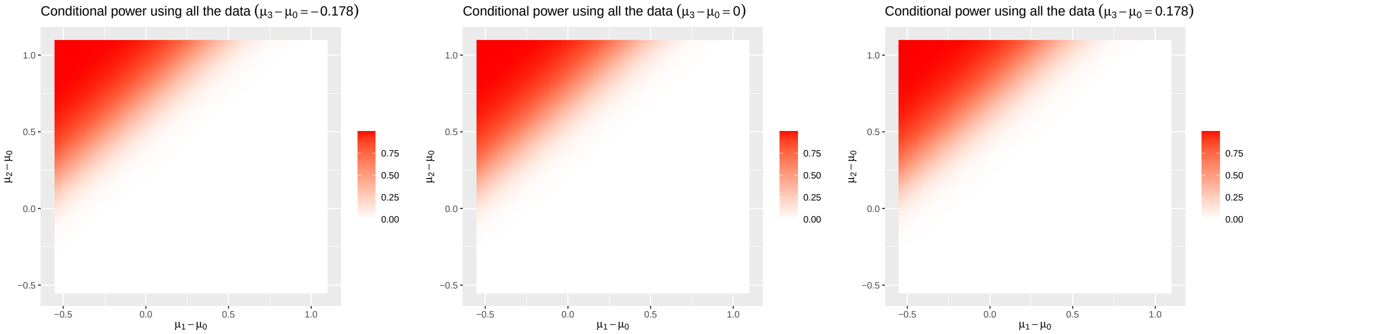

Web Figure 1. For multiple values of $\mu_3 - \mu_0$: the conditional power for treatment 2 given that treatment 1 has gone forward at the first stage when all the data is retained.

and

$$\Sigma^{\Xi_{k,2}} = \begin{pmatrix} 1 & \sqrt{\frac{1}{2}} & \frac{1}{2} & -\frac{1}{2}\sqrt{\frac{1}{2}} & \frac{1}{2} & -\frac{1}{2}\sqrt{\frac{1}{2}} \\ \sqrt{\frac{1}{2}} & 1 & \frac{1}{2}\sqrt{\frac{1}{2}} & -\frac{1}{2} & \frac{1}{2}\sqrt{\frac{1}{2}} & -\frac{1}{2} \\ \frac{1}{2} & \frac{1}{2}\sqrt{\frac{1}{2}} & 1 & \frac{1}{2}\sqrt{\frac{1}{2}} & \frac{1}{2} & 0 \\ -\frac{1}{2}\sqrt{\frac{1}{2}} & -\frac{1}{2} & \frac{1}{2}\sqrt{\frac{1}{2}} & 1 & 0 & \frac{1}{2} \\ \frac{1}{2} & \frac{1}{2}\sqrt{\frac{1}{2}} & \frac{1}{2} & 0 & 1 & \frac{1}{2}\sqrt{\frac{1}{2}} \\ -\frac{1}{2}\sqrt{\frac{1}{2}} & -\frac{1}{2} & 0 & \frac{1}{2} & \frac{1}{2}\sqrt{\frac{1}{2}} & 1 \end{pmatrix}.$$

We have $\Omega_{k^\star,k',1}$ equals Equation (Web Appendix E.2) and $\Omega^\star_{k^\star,k',1}$ equals Equation (Web Appendix E.1) multiplied by Equation (Web Appendix E.3).

Web Appendix G The effect of different values of $\mu_3 - \mu_0$ for the motivating trial example

The results of using different values of $\mu_3 - \mu_0$ are studied. The values studied are $\mu_3 - \mu_0 = -\theta_0$, $\mu_3 - \mu_0 = 0$ and $\mu_3 - \mu_0 = \theta_0$. The conditional power for treatment 2 given treatment 1 has become the new control at stage 1 when using all the data is given in Figure 1. The conditional power for treatment 2 given treatment 1 has become the new control at stage 1 when using only the new data is given in Figure 2. The difference in conditional power for treatment 2 given treatment 1 has become the new control at stage 1 is given in Figure 3. One can see the conditional type I error in these figures as well, for any points $\mu_2 \leq \mu_1$. The overall power when using all the data is given in Figure 4. The overall power when using only the new data is given in Figure 5. The difference in overall power is given in Figure 6. The probability of the treatment which does not have the greatest treatment effect becoming the control at the first stage is given in Figure 7.

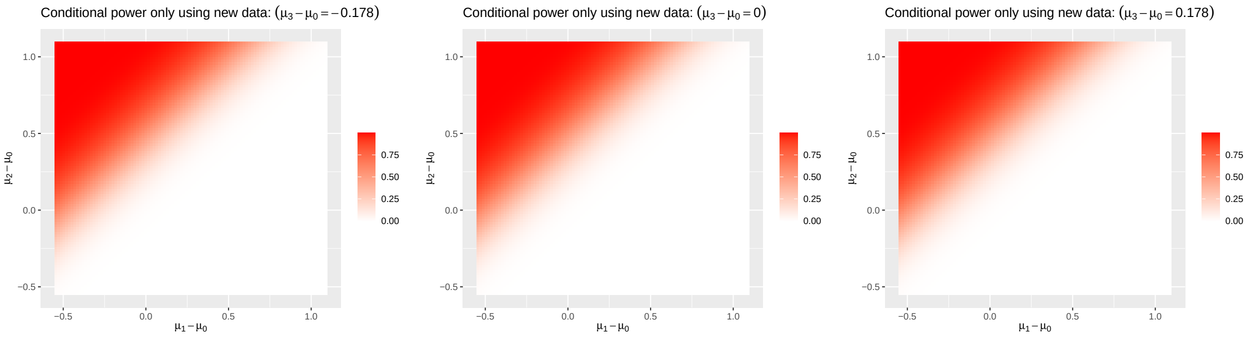

Web Figure 2. For multiple values of $\mu_3 - \mu_0$: the conditional power for treatment 2 given that treatment 1 has gone forward at the first stage when only the data post the change in control is used.

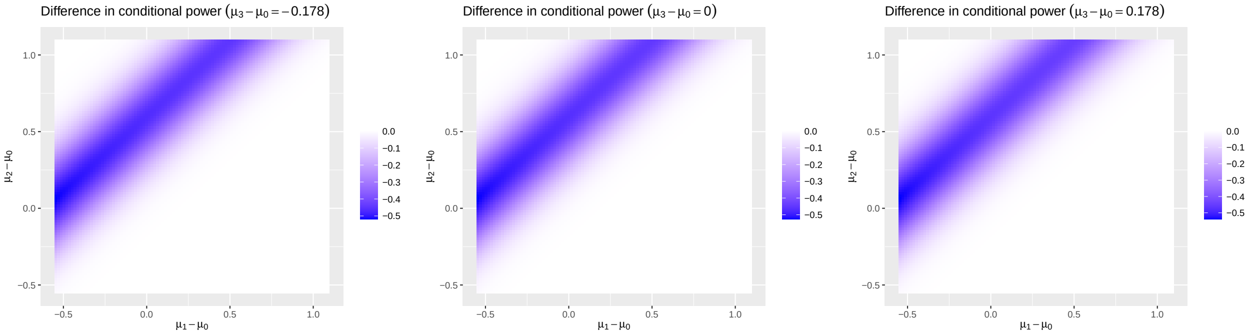

Web Figure 3. For multiple values of $\mu_3 - \mu_0$: the difference in conditional power between keeping the data pre change and not.

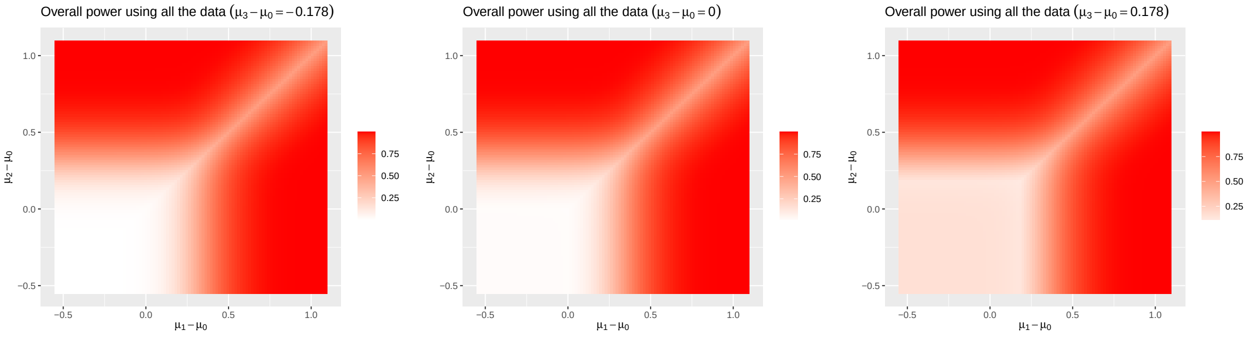

Web Figure 4. For multiple values of $\mu_3 - \mu_0$: the overall power when all the data is retained.

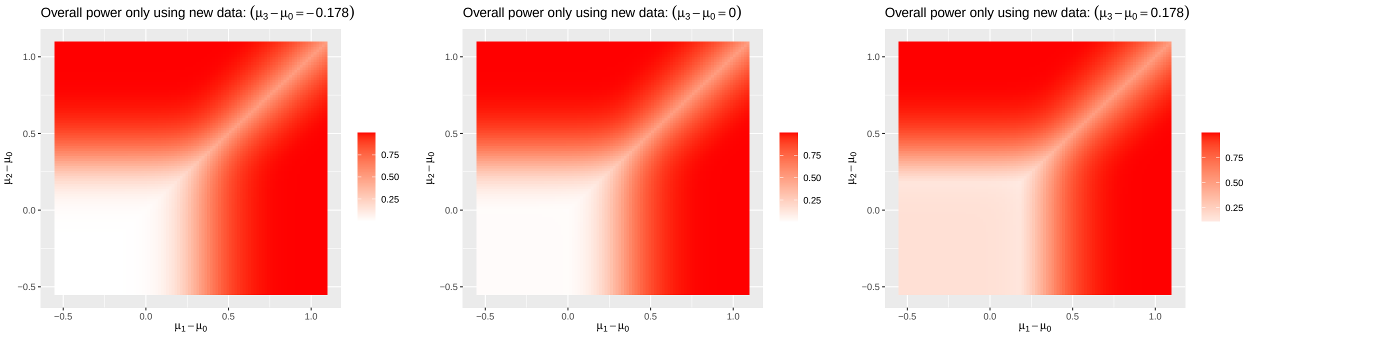

Web Figure 5. For multiple values of $\mu_3 - \mu_0$: the overall power when only the data post the change in control is used.

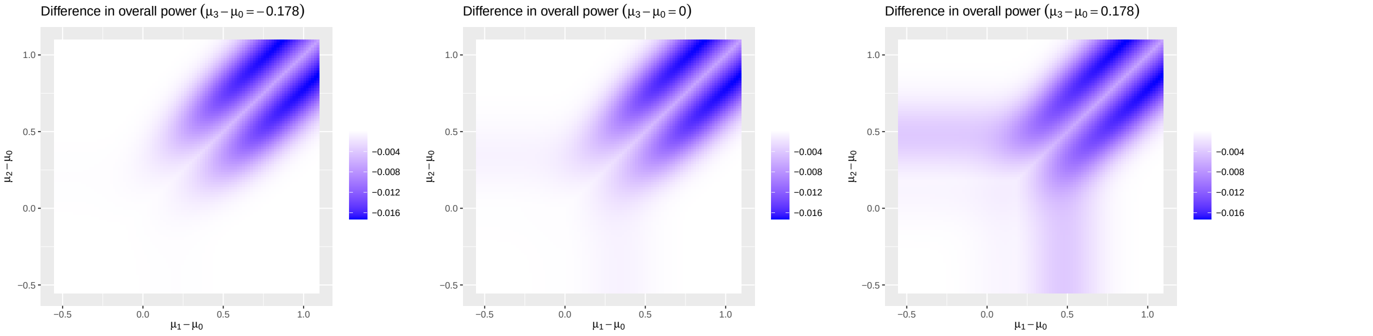

Web Figure 6. For multiple values of $\mu_3 - \mu_0$: the difference in overall power between keeping the data pre change and not.

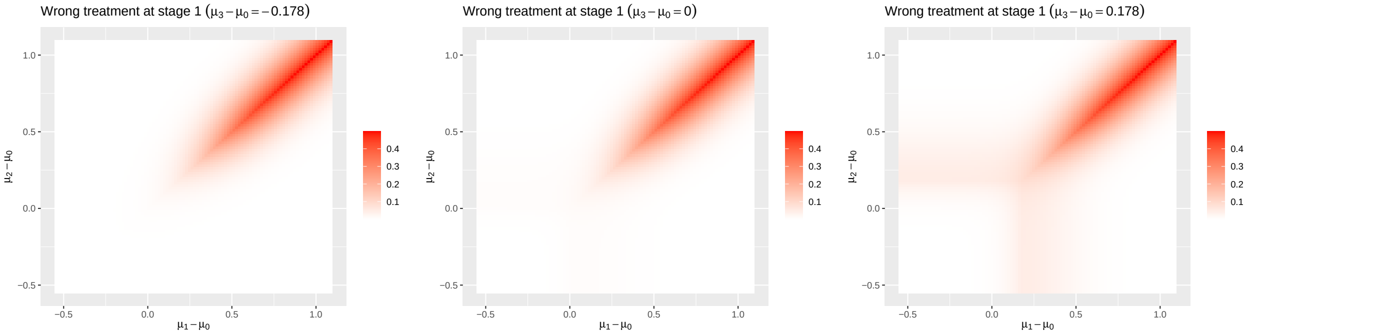

Web Figure 7. For multiple values of $\mu_3 - \mu_0$: the probability of the treatment which does not have the greatest treatment effect becoming the control at the first stage.

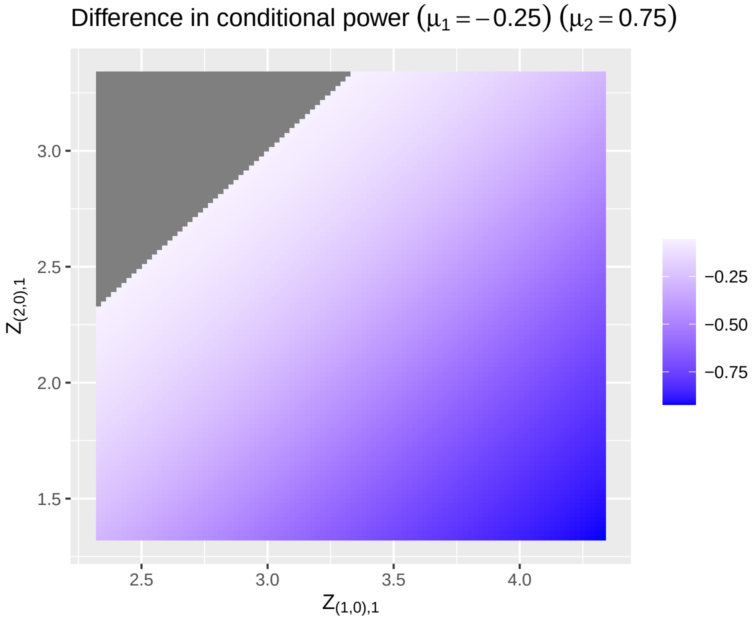

Web Figure 8. The difference in conditional power based on the value of the test statistics for treatment 2 given that treatment 1 has gone forward at the first stage for fixed $\mu_1 = -0.25$ and $\mu_2 = 0.75$.

Web Appendix H The effect of different values of $Z_{(1,0),1}$ and $Z_{(2,0),1}$ on the conditional power for given μ_1 and μ_2

Figure 8 shows the effect on conditional power for treatment 2 given treatment 1 became the control after the first stage, for different possible values of $Z_{(1,0),1}$ and $Z_{(2,0),1}$ given that $\mu_1 = -0.25$ and $\mu_2 = 0.75$. The grey area is the values of $Z_{(1,0),1}$ and $Z_{(2,0),1}$ which are not possible as $Z_{(1,0),1} < Z_{(2,0),1}$. One only needs to consider values of $Z_{(1,0),1} > u_1$ as otherwise treatment 1 would not be the new control. It is shown here that even in a case where on average there is very little benefit in only retaining the new information there are potential values of $Z_{(1,0),1}$ and $Z_{(2,0),1}$ where there is large benefit in only using the new data. For example if $Z_{(1,0),1} = 4$ and $Z_{(2,0),1} = 1.5$ then there is an loss in conditional power of 82.7% by retaining the old data.

Web Appendix I Three stage conditional power given change after the first stage

The only conditional power where one may see benefit in keeping the old data in a 3 stage case when $u_j > 0$ for all j is if the control changes at the first stage. For this example, due to each treatment getting the same number of patients per stage we define $n = n_1$ therefore the number of patients recruited at the second stage is $2n$, and the number of patients recruited at the third stage is $3n$. The denominator of the conditional power for a change after the first

stage is

$$P(E^1_{k^\star,k',1} \cap E^2_{k^\star,k',1} \cap E^3_{k^\star,k',1}) = \int_{u_1}^{\infty}\int_{l_1}^{\infty}\int_{-\infty}^{0}\int_{-\infty}^{0} \phi\Big(\mathbf{z}, \mu^{\Omega_{k^\star,k',1}}_{[1,2,3,4]}, \Sigma^{\Omega_{k^\star,k',1}}_{[1,2,3,4]}\Big)\mathbf{dz},$$

where $\phi(\mathbf{z}, \mu, \Sigma)$ is the probability density function of a multi-variate normal distribution with mean μ and covariance matrix Σ. Also

$$\mu^{\Omega_{k^\star,k',1}} = \Big\{\frac{\sqrt{n}(\mu_{k'} - \mu_0)}{\sigma\sqrt{2}}, \frac{\sqrt{n}(\mu_{k^\star} - \mu_0)}{\sigma\sqrt{2}}, \frac{\sqrt{n}(\mu_{k^\star} - \mu_{k'})}{\sigma\sqrt{2}}, \frac{\sqrt{n}(\mu_{k} - \mu_{k'})}{\sigma\sqrt{2}}, \frac{\sqrt{n}(\mu_{k^\star} - \mu_{k'})}{\sigma}, \frac{\sqrt{3n}(\mu_{k'} - \mu_0)}{\sigma\sqrt{2}}\Big\},$$

and

$$\Sigma^{\Omega_{k^\star,k',1}} = \begin{pmatrix} 1 & \frac{1}{2} & -\frac{1}{2} & -\frac{1}{2} & -\frac{1}{2}\sqrt{\frac{1}{2}} & -\frac{1}{2}\sqrt{\frac{1}{3}} \\ \frac{1}{2} & 1 & \frac{1}{2} & 0 & \frac{1}{2}\sqrt{\frac{1}{2}} & \frac{1}{2}\sqrt{\frac{1}{3}} \\ -\frac{1}{2} & \frac{1}{2} & 1 & \frac{1}{2} & \sqrt{\frac{1}{2}} & \sqrt{\frac{1}{3}} \\ -\frac{1}{2} & 0 & \frac{1}{2} & 1 & \frac{1}{2}\sqrt{\frac{1}{2}} & \frac{1}{2}\sqrt{\frac{1}{3}} \\ -\frac{1}{2}\sqrt{\frac{1}{2}} & \frac{1}{2}\sqrt{\frac{1}{2}} & \sqrt{\frac{1}{2}} & \frac{1}{2}\sqrt{\frac{1}{2}} & 1 & \sqrt{\frac{2}{3}} \\ -\frac{1}{2}\sqrt{\frac{1}{3}} & \frac{1}{2}\sqrt{\frac{1}{3}} & \sqrt{\frac{1}{3}} & \frac{1}{2}\sqrt{\frac{1}{3}} & \sqrt{\frac{2}{3}} & 1 \end{pmatrix}.$$

The numerator of the conditional power for a change after the first stage is

$$P(E^1_{k^\star,k',1} \cap E^2_{k^\star,k',1} \cap E^3_{k^\star,k',1} \cap E^4_{k^\star,k',1}) = \int_{u_1}^{\infty}\int_{l_1}^{\infty}\int_{-\infty}^{0}\int_{-\infty}^{0}\int_{u_2}^{\infty} \phi\Big(\mathbf{z}, \mu^{\Omega_{k^\star,k',1}}_{[1,2,3,4,5]}, \Sigma^{\Omega_{k^\star,k',1}}_{[1,2,3,4,5]}\Big)\mathbf{dz} + \int_{u_1}^{\infty}\int_{l_1}^{\infty}\int_{-\infty}^{0}\int_{-\infty}^{0}\int_{l_2}^{u_2}\int_{u_3}^{\infty} \phi\Big(\mathbf{z}, \mu^{\Omega_{k^\star,k',1}}, \Sigma^{\Omega_{k^\star,k',1}}\Big)\mathbf{dz}.$$

The conditional power for treatment $k^\star$ when treatment k' becomes the new control at stage 1 is

$$\frac{P(E^1_{k^\star,k',1} \cap E^2_{k^\star,k',1} \cap E^3_{k^\star,k',1} \cap E^4_{k^\star,k',1})}{P(E^1_{k^\star,k',1} \cap E^2_{k^\star,k',1} \cap E^3_{k^\star,k',1})}.$$

When we only retain the new information the conditional power is

$$P(E^{\star 4}_{k^\star,k',1}) = \int_{u_2}^{\infty} \phi\Big\{\mathbf{z}, \frac{\sqrt{n}(\mu_{k^\star} - \mu_{k'})}{\sigma\sqrt{2}}, 1\Big\}\mathbf{dz}.$$

The effect of using all the data; the post change data and the difference for conditional power between keeping the old data and not for the O'Brien and Fleming bounds (O'Brien and Fleming, 1979) are given in Figure 9, 10 and 11 respectively. The O'Brien and Fleming bounds are $u_1 = 3.640, u_2 = 2.574, u_3 = 2.101$ and $l_1 = -3.640, l_2 = -2.574, l_3 = 2.101$ found using the (Magirr et al., 2012). The maximum sample size using Greenstreet et al. (2025) is 312 which is based on 26 patients per stage per arm.

As can be seen even when using the negative bounds given by O'Brien and Fleming there is no advantage to keeping the old data in this example. This means that in this case for the overall

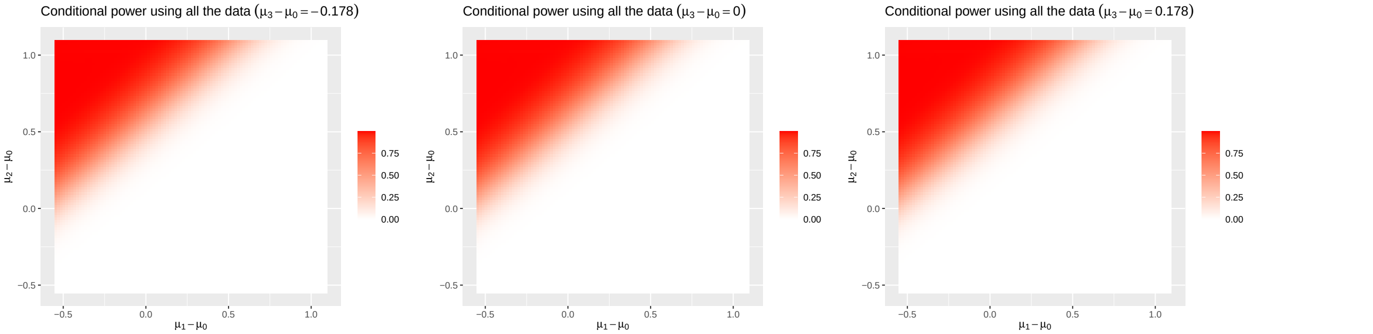

Web Figure 9. For multiple values of $\mu_3 - \mu_0$ for the 3 stage example: the conditional power for treatment 2 given that treatment 1 has gone forward at the first stage when all the data is retained.

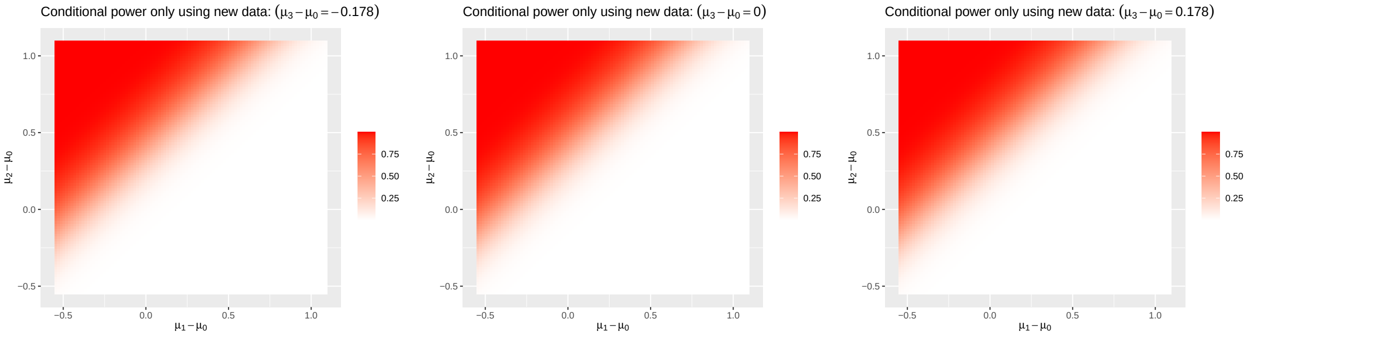

Web Figure 10. For multiple values of $\mu_3 - \mu_0$ for the 3 stage example: the conditional power for treatment 2 given that treatment 1 has gone forward at the first stage when only the new data is retained.

power there is also no benefit to retaining the old data. However in the Supporting Information Section Web Appendix J we study a 3 stage example where there is benefit in keeping the old data when there are negative bounds.

Web Appendix J Example where negative bounds can cause loss in power when only keeping new data

Consider a three stage example with $n_1 = 100$ $n_2 = 101$ and $n_3 = 10000$ and the lower bounds $l_1 = l_2 = -1.1$ and $u_1 = u_2 = u_3 = 2$. We set $\mu_1 = 1000$, $\mu_2 = 1000.3$, $\mu_3 = \mu_0 = 0$. Then similar to above we focus on the conditional power of treatment 2 given treatment 1 has gone forward at the first stage. The conditional power is 96.4% when data is retained compared to 90.5% when only the new data is retained. This is because after the first stage there is a 9.47% chance that treatment 2 is dropped compared to treatment 1 when only the new data is used. However when old data is kept this drops to 3.63%. This therefore makes it more likely that treatment 2 will get to the final stage where there is a very high chance it will now be found superior to the control.

However even for this example if one changes the difference of $\mu_1 - \mu_2$ then one will likely find

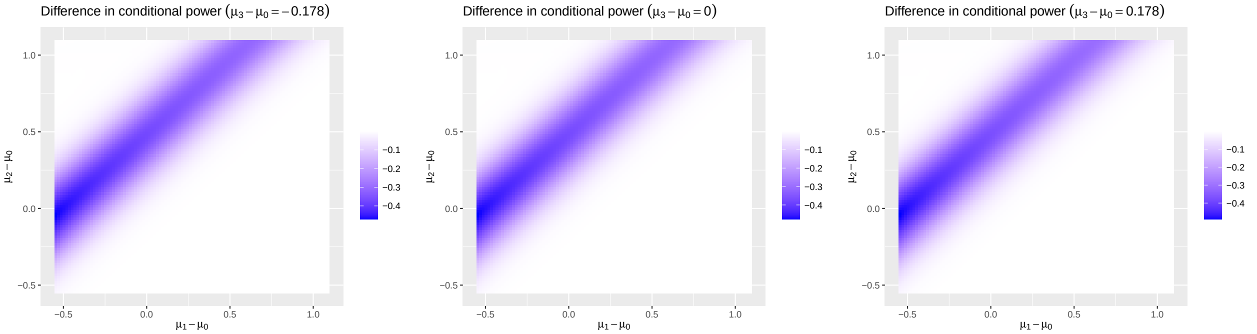

Web Figure 11. For multiple values of $\mu_3 - \mu_0$ for the 3 stage example: the difference in conditional power between keeping the data pre change and not.

that keeping the old data is worse than not. This section highlights that it is possible when there are negative bounds that keeping the old data can be positive, however this may not be very likely. As one can show that the conditional power difference can be positive for keeping the data, this means that this also holds for the overall power.

Web Appendix K Simple random allocation effect on conditional power

We use a simple random allocation method to study the effect on conditional power. We simulate 100,000,000 runs of the case where treatment 1 has been taken forward after the first stage and treatment 2 is the treatment of interest. It is assumed that $\mu_3 - \mu_0 = 0$ for the example and the triangular boundaries are used as defined in Section 5. The results of 3 different cases are given in Table 1. As can be seen in Table 1 when $\mu_1 - \mu_0 = 0.178$ and $\mu_2 - \mu_0 = 0.545$ the conditional power is still a lot higher when using only the data post change in control. Furthermore for this example there were only 167 simulations that found keeping all the data resulted in treatment 2 being found superior, when it was not found superior when using only the new data. This is compared to 26165252 for the other way round. This has highlighted that the conditional power and therefore overall power is still likely less when all the data is retained even when using a simple random allocation method.

Web Table 1. The conditional power when using a simple random allocation

Treatment effects		Conditional power		Treatment 2 only goes forward	
$\mu_1 = \mu_0$	$\mu_2 = \mu_0$	new data	all data	with new data	with all data
0.545	1.090	61,21%	19.37%	41832417	649
0.545	0.723	8.26%	0.39%	7870200	18
0.178	0.545	30.01%	3.84%	26165252	167

Web Appendix L Complete equations for calculating conditional and overall power when adding additional arms

Web Appendix L.1 Notation

Consider a clinical trial with up to K experimental arms that will be tested against one common control arm. Let $n_{k,j}$ denote the number of patients recruited to treatment k by the end of its jth stage assuming that recruitment of this arm had begun at the start.

The number of patients recruited between interim analyses is equal i.e. $n_{k,j} - n_{k,j^\star} = n_{k^\star,j} - n_{k^\star,j^\star}$ for all $k, k^\star \in \{0, \dots, K\}$ and $j, j^\star \in \{0, \dots, J\}$. Let $n_{k,0}$ define the number of patients already recruited to an active treatment that started the trial before treatment k enters the trial. We have $k'_{n_{k',j'}}$ denoting the current control treatment at point $n_{k',j'}$, where j' is the stage for treatment k' where it became the control, with $k' \in \{0, \dots, K\}$ and $j' \in \{0, \dots, J\}$. Therefore $n_{k',j'}$ denotes the number of patients recruited prior to treatment k' becoming control at its j' th stage. For simplicity we drop the subscript from $k'_{n_{k',j'}}$ as the focus of this work will be on only changing the control group once, with, $k' = 0$ at the beginning of the trial.

Each of the K hypotheses is potentially tested at a series of analyses indexed by $j \in \{\ddot{j}_{k,k',j'} + 1, \dots, J\}$ where $\ddot{j}_{k,k',j'}$ is the last stage for k before k' became the control. When all the treatments begin at once then $\ddot{j}_{k,k',j'} = j'$ as each interim for each treatment happens at the same time. However if treatments are added at different points this may not be the case. For example in a 3 arm trial if treatment 1 becomes the control at its first stage and treatment 2 is not added till after treatment 1's first analysis then $\ddot{j}_{2,1,1} = 0$. At analysis j for treatment k, to test $H_{k'k}$ it is assumed that responses, $X_{k,i}$ and $X_{k',i'}$, from patients $i = n_{k,0}, \dots, n_{k,j}$ and $i' = n_{k',0}, \dots, n_{k,j}$ are observed respectively. These hypotheses are tested at given analysis j using the test statistic

$$Z_{k,k',j} = \frac{\sum_{i=\max(n_{k,0},n_{k',0})+1}^{n_{k,j}} X_{k,i} - \sum_{i=\max(n_{k,0},n_{k',0})+1}^{n_{k,j}} X_{k',i}}{\sigma\sqrt{2(n_{k,j} - \max(n_{k,0}, n_{k',0}))}}.$$

To ensure only concurrent controls are used we have $\max(n_{k,0}, n_{k',0})$. If only the data post the change in the control is used the test statistics are

$$Z^\star_{k,k',j,j'} = \frac{\sum_{i=\max(n_{k,0},n_{k',0},n_{k',j'})+1}^{n_{k,j}} X_{k,i} - \sum_{i=\max(n_{k,0},n_{k',0},n_{k',j'})+1}^{n_{k,j}} X_{k',i}}{\sigma\sqrt{2(n_{k,j} - \max(n_{k,0}, n_{k',0}, n_{k',j'}))}},$$

where $\max(n_{k,0}, n_{k',0}, n_{k',j'})$ includes the point in which the control changes and only if $n_{k,0}, n_{k',0} \leq n_{k',j'}$ will $Z^\star_{k,k',j,j'} \neq Z_{k,k',j}$.

Web Appendix L.2 Conditional power and conditional type I error

The conditional power for a given treatment $k^\star$ is the probability that given treatment k' is the new standard of care after its j'^{th} stage that treatment $k^\star$ is found superior to the new control

k', when tested for treatment $k^\star$ remaining analyses. The conditional type I error is the same given that $\mu_{k^\star} \leq \mu_{k'}$.

In order to equate the conditional power one can use the conditional probability definition to remove the need to calculate any highly truncated normal distributions. The conditional power is

$$\begin{cases} 0 & \text{if } n_{k^\star,J} \leq n_{k',j'} \\ \frac{P(E^1_{k^\star,k',j'} \cap E^2_{k^\star,k',j'} \cap E^3_{k^\star,k',j'} \cap E^4_{k^\star,k',j'})}{P(E^1_{k^\star,k',j'} \cap E^2_{k^\star,k',j'} \cap E^3_{k^\star,k',j'})} & \text{if } n_{k^\star,J} > n_{k',j'} \end{cases}.$$

The event $E^1_{k^\star,k',j'}$ which is the event that treatment k' becomes the control at it's j'^{th} stage equals,

$$E^1_{k^\star,k',j'} = \bigcap_{i=1}^{j'-1} (l_i \leq Z_{k',0,i} \leq u_i) \cap Z_{k',0,j'} \geq u_{j'}.$$

The event $E^2_{k^\star,k',j'}$ which is that treatment $k^\star$ is still in the trial when treatment k' becomes the control equals,

$$\begin{aligned} E^2_{k^\star,k',j'} =& (n_{k^\star,1} > n_{k',j'}) \cup (n_{k^\star,1} \leq n_{k',j'}) \cap \Bigg(\Bigg[(n_{k^\star,\ddot{j}_{k^\star,k',j'}} < n_{k',j'}) \cap \\ & \bigcap_{i=1}^{\ddot{j}_{k^\star,k',j'}} (l_i \leq Z_{k^\star,0,i} \leq u_i) \Bigg] \cup \Bigg[(n_{k^\star,\ddot{j}_{k^\star,k',j'}} = n_{k',j'}) \bigcap_{i=1}^{\ddot{j}_{k^\star,k',j'}-1} (l_i \leq Z_{k',0,i} \leq u_i) \cap \\ & (l_{\ddot{j}_{k^\star,k',j'}} \leq Z_{k^\star,0,\ddot{j}_{k^\star,k',j'}}) \cap \{ (Z_{k^\star,0,\ddot{j}_{k^\star,k',j'}} \leq u_{\ddot{j}_{k^\star,k',j'}}) \cup (Z_{k^\star,k,\ddot{j}_{k^\star,k',j'}} \leq 0) \} \Bigg] \Bigg(\end{aligned}$$

The event $E^3_{k^\star,k',j'}$ which is that none of the other k treatments become the control is

$$\begin{aligned} E^3_{k^\star,k',j'} = & \bigcap_{k \in (1 \ldots K)/k^\star,k'} \Bigg[(n_{k,1} > n_{k',j'}) \cup (n_{k,1} \leq n_{k',j'}) \cap \\ & \Bigg\{ \Bigg[\bigcup_{i=1}^{\ddot{j}_{k,k',j'}-1} \bigcap_{i^\star=1}^{i-1} (l_{i^\star} \leq Z_{k,0,i^\star} \leq u_{i^\star}) \cap (Z_{k,0,i} \leq l_i) \Bigg] \cup \Bigg(\Bigg[(n_{k,\ddot{j}_{k,k',j'}} < n_{k',j'}) \\ & \cap \bigcap_{i=1}^{\ddot{j}_{k,k',j'}-1} (l_i \leq Z_{k,0,i} \leq u_i) \cap (Z_{k,0,\ddot{j}_{k,k',j'}} \leq u_{\ddot{j}_{k,k',j'}}) \Bigg] \cup \Bigg[(n_{k,\ddot{j}_{k,k',j'}} = n_{k',j'}) \cap \\ & \bigcap_{i=1}^{\ddot{j}_{k,k',j'}-1} (l_i \leq Z_{k,0,i} \leq u_i) \cap \{ (Z_{k,0,\ddot{j}_{k,k',j'}} \leq u_{\ddot{j}_{k,k',j'}}) \cup (Z_{k,k',\ddot{j}_{k,k',j'}} < 0) \} \Bigg] \Bigg) \Bigg\} \Bigg]. \end{aligned}$$

The event $E^4_{k',k^\star,j'}$ which is the event that we reject $H_{k'k}$ within the rest of the trial equals,

$$E^4_{k^\star,k',j'} = \bigcup_{i=\ddot{j}_{k^\star,k',j'}+1}^{J} \bigcap_{i^\star=\ddot{j}_{k^\star,k',j'}+1}^{i-1} (l_{i^\star} \leq Z_{k^\star,k',i^\star} \leq u_{i^\star}) \cap (u_i < Z_{k^\star,k',i}).$$

The event $E^{\star 4}_{k^\star,k',j'}$ which is the event that we reject $H_{k'k}$ within the rest of the trial when not retaining the information post the change in control treatment equals,

$$E^{\star 4}_{k^\star,k',j'} = \bigcup_{i=\ddot{j}_{k^\star,k',j'}+1}^{J} \bigcap_{i^\star=\ddot{j}_{k^\star,k',j'}+1}^{i-1} (l_{i^\star} \leq Z^\star_{k^\star,k',i^\star,j'} \leq u_{i^\star}) \cap (u_i < Z^\star_{k^\star,k',i,j'}).$$

The conditional power can be calculated using multivariate normal distributions using the mean of each test statistic $Z_{k,k',j}$,

$$\frac{(\mu_k - \mu_{k'})\sqrt{(n_{k,j} - \max(n_{k,0}, n_{k',0}))}}{\sigma\sqrt{2}},$$

and the correlation matrix, Σ. The correlation matrix can be split into multiple values, $\psi_{(k_1,k_1',j_1),(k_2,k_2',j_2)}$, that depend on the correlation between Z_{k_1,k_1',j_1} and Z_{k_2,k_2',j_2}, and $\psi_{(k_1,k_1',j_1),(k_2,k_2',j_2)}$ equals,

$$\begin{cases} 0 & \text{for } k_1 \neq k_2, k_2' \text{ \& } k_1' \neq k_2, k_2' \\ \frac{\max(0, n_{k_1,j_1} - \max(n_{k_1,0}, n_{k_1',0}, n_{k_2',0}))}{2\sqrt{(n_{k_1,j_1} - \max(n_{k_1,0}, n_{k_1',0}))(n_{k_1,j_2} - \max(n_{k_1,0}, n_{k_2',0}))}} & \text{for } k_1 = k_2 \text{ \& } k_1' \neq k_2' \text{ \& } n_{k_1,j_1} \leq n_{k_2,j_2} \\ -\frac{\max(0, n_{k_1,j_1} - \max(n_{k_1,0}, n_{k_2,0}, n_{k_1',0}))}{2\sqrt{(n_{k_1,j_1} - \max(n_{k_1,0}, n_{k_1',0}))(n_{k_2,j_2} - \max(n_{k_2,0}, n_{k_1,0}))}} & \text{for } k_1 = k_2' \text{ \& } k_1' \neq k_2 \text{ \& } n_{k_1,j_1} \leq n_{k_2,j_2} \\ \frac{\max(0, n_{k_1,j_1} - \max(n_{k_1,0}, n_{k_2,0}, n_{k_1',0}))}{2\sqrt{(n_{k_1,j_1} - \max(n_{k_1,0}, n_{k_1',0}))(n_{k_2,j_2} - \max(n_{k_2,0}, n_{k_1',0}))}} & \text{for } k_1 \neq k_2 \text{ \& } k_1' = k_2' \text{ \& } n_{k_1,j_1} \leq n_{k_2,j_2} \\ \sqrt{\frac{n_{k_1,j_1} - \max(n_{k_1,0}, n_{k_1',0})}{n_{k_1,j_2} - \max(n_{k_1,0}, n_{k_1',0})}} & \text{for } k_1 = k_2 \text{ \& } k_1' = k_2' \text{ \& } n_{k_1,j_1} \leq n_{k_2,j_2}. \end{cases}$$

In the case of only considering the data post changing the control, the test statistics before the change are now independent of the test statistics post the change. Therefore one only needs the event that we reject $H_{k'k}$ within the rest of the trial. For the case where only the post change data is used we define this as $E^{\star 4}_{k^\star,k',j'}$.

$$E^{\star 4}_{k^\star,k',j'} = \bigcup_{i=\ddot{j}_{k^\star,k',j'}+1}^{J} \bigcap_{i^\star=\ddot{j}_{k^\star,k',j'}+1}^{i-1} (l_{i^\star} \leq Z^\star_{k^\star,k',i^\star,j'} \leq u_{i^\star}) \cap (u_i < Z^\star_{k^\star,k',i,j'}).$$

If treatment $k^\star$ joins the trial after treatment k' becomes the control then $E^4_{k^\star,k',j'} = E^{\star 4}_{k^\star,k',j'}$ as there is no data pre the change that is shared. The conditional power in this case is

$$\begin{cases} 0 & \text{if } n_{k,J} \leq n_{k',j'} \\ P(E^{\star 4}_{k^\star,k',j'}) & \text{if } n_{k,J} > n_{k',j'} \end{cases}.$$

Once again this can be calculated using multivariate normal distributions using the mean of each test statistic $Z_{k,k',j}$,

$$\frac{(\mu_k - \mu_{k'})\sqrt{(n_{k,j} - \max(n_{k,0}, n_{k',0}, n_{k',j'}))}}{\sigma\sqrt{2}};$$

and the correlation matrix which can be split into multiple $\psi_{i,i^\star}$ that depend on the correlation between $Z^\star_{k,k',j_1,j'}$ and $Z^\star_{k,k',j_2,j'}$, and equals,

$$\psi_{i,i^\star} = \begin{cases} \sqrt{\frac{n_{k,j_1} - \max(n_{k,0}, n_{k',j'})}{n_{k,j_2} - \max(n_{k,0}, n_{k',j'})}} & \text{for } j_1 \leq j_2. \end{cases}$$

Web Appendix L.3 Overall power

The overall power can be split into multiple, easy to compute, parts. The first of these is the probability that at each interim $j^\star$, treatment $k^\star$ becomes the control ($\Xi_{k^\star,j^\star}$) and this equals:

$$\Xi_{k^\star,j^\star} = P(E^1_{k^\star,k^\star,j^\star} \cap E^3_{k^\star,k^\star,j^\star}). \qquad \text{(Web Appendix L.1)}$$

The probability another treatment becomes the new control and then this treatment is found to be better then the new control ($\Omega_{k^\star,k',j'}$) can be split into every possible k' and j'.

$$\Omega_{k^\star,k',j'} = \begin{cases} 0 & \text{if } n_{k,J} \leq n_{k',j'} \\ P(E^1_{k',k^\star,j'} \cap E^2_{k',k^\star,j'} \cap E^3_{k',k^\star,j'} \cap E^4_{k',k^\star,j'}) & \text{if } n_{k,J} > n_{k',j'}. \end{cases} \qquad \text{(Web Appendix L.2)}$$

Combining Equation (Web Appendix L.1) and Equation (Web Appendix L.2) the overall power is

$$\sum_{j^\star=1}^{J} \Xi_{k^\star,j^\star} + \sum_{k'\in\{1,\dots,K\}/k^\star} \sum_{j'=1}^{J} \Omega_{k',k^\star,j'}.$$

When we consider only using the data post change in control the probability another treatment becomes the new control and then this treatment is found to be better then the new control ($\Omega^\star_{k^\star,k',j'}$) becomes

$$\Omega^\star_{k^\star,k',j'} = \begin{cases} 0 & \text{if } n_{k,J} \leq n_{k',j'} \\ P(E^1_{k^\star,k',j'} \cap E^2_{k^\star,k',j'} \cap E^3_{k^\star,k',j'})P(E^{\star 4}_{k^\star,k',j'}) & \text{if } n_{k,J} < n_{k',j'} \end{cases}.$$

This is due to the independence of event 4 with the rest of the events. Therefore the overall power is

$$\sum_{j^\star=1}^{J} \Xi_{k^\star,j^\star} + \sum_{k'\in\{1,\dots,K\}/k^\star} \sum_{j'=1}^{J} \Omega^\star_{k^\star,k',j'}.$$

Web Appendix L.4 Motivating trial example

We consider the motivating trial of TAILoR (Pushpakom et al., 2020). The TAILoR trial was a 4 arm trial which studied the effect of different doses of a treatment on HIV. The study had 1 interim analysis. We are going to use the operating characteristics from this study to see the effects on overall and conditional power if the control was changed mid trial if a treatment was found superior. In the original design the family wise error rate (FWER) (Pushpakom et al., 2015) was controlled at 5% one sided for a normal continuous endpoint and there was a planned 90% power. The trial was planned to have equal allocation across stages. In addition the clinically relevant effect of $\theta_1 = 0.545$ and uninteresting effect $\theta_0 = 0.178$ assuming the variance $\sigma^2 = 1$ was used.

Triangular stopping boundaries will be used (Whitehead, 1997) as recommended in Wason and Jaki (2012). We will consider the design if one of the treatments was added at the end of the first stage which is illustrated in Figure 12. Therefore the stopping boundaries will be found using the approach given in Greenstreet et al. (2024) to control FWER for the design before the change in control. The calculations of the power will be done using Greenstreet et al. (2025) to control the pairwise power for each treatment.

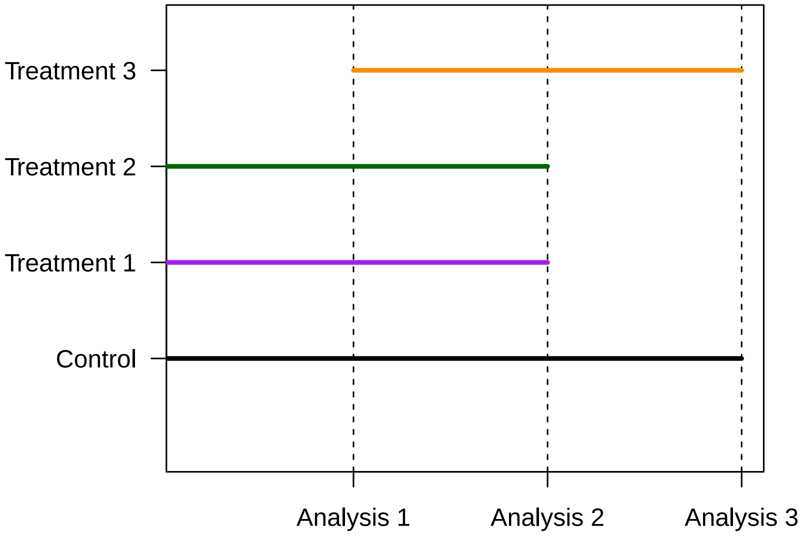

Web Figure 12. Illustration of the motivating trial when one treatment starts at the end of the first stage.

Web Appendix L.4.1 Boundaries and sample size

Using the approach by Greenstreet et al. (2024) the triangular stopping boundaries are found to be

$$U = \begin{pmatrix} u_1 & u_2 \end{pmatrix} = \begin{pmatrix} 2.358 & 2.223 \end{pmatrix}, \quad L = \begin{pmatrix} l_1 & l_2 \end{pmatrix} \begin{pmatrix} 0.786 & 2.223 \end{pmatrix}.$$

Based on 43 patients per arm per stage the maximum sample size is now 387 in order to control the pairwise power at 90% (Greenstreet et al., 2025). This addition accounts for the patients which would need to be added for the later treatment as seen in Figure 12. As each treatment gets the same number of treatments per stage we define $n = n_{k,1} - n_{k,0} = n_{k,2} - n_{k,1}$ for all $k \in \{0, 1, 2, 3\}$.

Web Appendix L.4.2 Conditional power and conditional type I error

The conditional power and conditional type I error for treatment 1 or 2 given the other has become the control at the first stage is calculated as follows:

$$P(E^1_{k^\star,k',1} \cap E^2_{k^\star,k',1} \cap E^3_{k^\star,k',1}) = \int_{u_1}^{\infty} \int_{l_1}^{\infty} \int_{-\infty}^{0} \phi\left(\mathbf{z}, \mu_{[1,2,3]}^{\Omega_{k^\star,k',1}}, \Sigma_{[1,2,3]}^{\Omega_{k^\star,k',1}}\right) \mathbf{dz},$$

and

$$P(E^1_{k^\star,k',1} \cap E^2_{k^\star,k',1} \cap E^3_{k^\star,k',1} \cap E^4_{k^\star,k',1}) = \int_{u_1}^{\infty} \int_{l_1}^{\infty} \int_{-\infty}^{0} \int_{u_2}^{\infty} \phi\left(\mathbf{z}, \mu^{\Omega_{k^\star,k',1}}, \Sigma^{\Omega_{k^\star,k',1}}\right) \mathbf{dz},$$

where

$$\mu^{\Omega_{k^\star,k',1}} = \left\{ \frac{\sqrt{n}(\mu_{k'}-\mu_0)}{\sigma\sqrt{2}}, \frac{\sqrt{n}(\mu_{k^\star}-\mu_0)}{\sigma\sqrt{2}}, \frac{\sqrt{n}(\mu_{k^\star}-\mu_{k'})}{\sigma\sqrt{2}}, \frac{\sqrt{n}(\mu_{k^\star}-\mu_{k'})}{\sigma} \right\},$$

and

$$\Sigma^{\Omega_{k^\star,k',1}} = \begin{pmatrix} 1 & \frac{1}{2} & -\frac{1}{2} & -\frac{1}{2}\sqrt{\frac{1}{2}} \\ \frac{1}{2} & 1 & \frac{1}{2} & \frac{1}{2}\sqrt{\frac{1}{2}} \\ -\frac{1}{2} & \frac{1}{2} & 1 & \sqrt{\frac{1}{2}} \\ -\frac{1}{2}\sqrt{\frac{1}{2}} & \frac{1}{2}\sqrt{\frac{1}{2}} & \sqrt{\frac{1}{2}} & 1 \end{pmatrix}.$$

When only new data is kept the conditional power is:

$$P(E^{\star 4}_{k^\star,k',1}) = \int_{u_2}^{\infty} \phi\left\{\mathbf{z}, \frac{\sqrt{n}(\mu_{k^\star}-\mu_{k'})}{\sigma\sqrt{2}}, 1\right\}\mathbf{dz}.$$

The conditional power for treatment 3 given that either treatment 1 or 2 has become the new control at their first stage is calculated as follows.

$$P(E^1_{k^\star,k',1} \cap E^2_{k^\star,k',1} \cap E^3_{k^\star,k',1} \cap E^4_{k^\star,k',1}) = P(E^1_{k^\star,k',1} \cap E^2_{k^\star,k',1} \cap E^3_{k^\star,k',1})P(E^4_{k^\star,k',1}),$$

where $P(E^1_{k^\star,k',1} \cap E^2_{k^\star,k',1} \cap E^3_{k^\star,k',1})$ is equals

$$P(E^1_{k^\star,k',1} \cap E^2_{k^\star,k',1} \cap E^3_{k^\star,k',1}) = \int_{u_1}^{\infty}\int_{-\infty}^{0} \phi\left\{\mathbf{z}, \left(\frac{\sqrt{n}(\mu_{k'}-\mu_0)}{\sigma\sqrt{2}}, \frac{\sqrt{n}(\mu_{k^\star}-\mu_{k'})}{\sigma\sqrt{2}}\right), \begin{pmatrix} 1 & -\frac{1}{2} \\ -\frac{1}{2} & 1 \end{pmatrix} \right\}\mathbf{dz},$$

and $P(E^4_{k^\star,k',1})$ is

$$P(E^4_{k^\star,k',1}) = \int_{u_1}^{\infty} \phi\left\{\mathbf{z}, \frac{\sqrt{n}(\mu_{k^\star}-\mu_{k'})}{\sigma\sqrt{2}}, 1\right\}\mathbf{dz} + \int_{l_1}^{u_1}\int_{u_2}^{\infty} \phi\left\{\mathbf{z}, \left(\frac{\sqrt{n}(\mu_{k^\star}-\mu_{k'})}{\sigma\sqrt{2}}, \frac{\sqrt{n}(\mu_{k^\star}-\mu_{k'})}{\sigma}\right), \begin{pmatrix} 1 & \sqrt{\frac{1}{2}} \\ \sqrt{\frac{1}{2}} & 1 \end{pmatrix} \right\}\mathbf{dz}.$$

Therefore the conditional power for both when old concurrent data is used and only new concurrent data is used is $P(E^4_{k^\star,k',1})$.

When treatment 1 or treatment 2 becomes the control at their second stage the conditional power is as follows, where we define treatment $k_1 = \{1,2\}/k'$ for the other treatment tested

which did not become the control.

$$P(E^1_{k^\star,k',2} \cap E^2_{k^\star,k',2} \cap E^3_{k^\star,k',2}) = \int_{l_1}^{u_1}\int_{u_2}^{\infty}\int_{l_1}^{u_1}\int_{-\infty}^{l_1} \phi, \left(\mathbf{z}, \mu^{\Omega_{k^\star,k',2}}_{[1,2,3,5]}, \Sigma^{\Omega_{k^\star,k',2}}_{[1,2,3,5]}\right)\mathbf{dz}$$
$$+ \int_{l_1}^{u_1}\int_{u_2}^{\infty}\int_{l_1}^{u_1}\int_{l_1}^{u_1}\int_{-\infty}^{0} \phi\left(\mathbf{z}, \mu^{\Omega_{k^\star,k',2}}_{[1,2,3,5,6]}, \Sigma^{\Omega_{k^\star,k',2}}_{[1,2,3,5,6]}\right)\mathbf{dz}$$
$$+ \int_{l_1}^{u_1}\int_{u_2}^{\infty}\int_{u_1}^{\infty}\int_{-\infty}^{0}\int_{-\infty}^{l_1} \phi\left(\mathbf{z}, \mu^{\Omega_{k^\star,k',2}}_{[1,2,3,4,5]}, \Sigma^{\Omega_{k^\star,k',2}}_{[1,2,3,4,5]}\right)\mathbf{dz}$$
$$+ \int_{l_1}^{u_1}\int_{u_2}^{\infty}\int_{u_1}^{\infty}\int_{-\infty}^{0}\int_{l_1}^{u_1}\int_{-\infty}^{0} \phi\left(\mathbf{z}, \mu^{\Omega_{k^\star,k',2}}_{[1,2,3,4,5,6]}, \Sigma^{\Omega_{k^\star,k',2}}_{[1,2,3,4,5,6]}\right)\mathbf{dz},$$

and

$$\Omega_{k^\star,k',2} = P(E^1_{k^\star,k',2} \cap E^2_{k^\star,k',2} \cap E^3_{k^\star,k',2} \cap E^4_{k^\star,k',2}) =$$
$$\int_{l_1}^{u_1}\int_{u_2}^{\infty}\int_{l_1}^{u_1}\int_{-\infty}^{l_1}\int_{u_2}^{\infty} \phi\left(\mathbf{z}, \mu^{\Omega_{k^\star,k',2}}_{[1,2,3,5,7]}, \Sigma^{\Omega_{k^\star,k',2}}_{[1,2,3,5,7]}\right)\mathbf{dz}$$
$$+ \int_{l_1}^{u_1}\int_{u_2}^{\infty}\int_{l_1}^{u_1}\int_{l_1}^{u_1}\int_{-\infty}^{0}\int_{u_2}^{\infty} \phi\left(\mathbf{z}, \mu^{\Omega_{k^\star,k',2}}_{[1,2,3,5,6,7]}, \Sigma^{\Omega_{k^\star,k',2}}_{[1,2,3,5,6,7]}\right)\mathbf{dz}$$
$$+ \int_{l_1}^{u_1}\int_{u_2}^{\infty}\int_{u_1}^{\infty}\int_{-\infty}^{0}\int_{-\infty}^{l_1}\int_{u_2}^{\infty} \phi\left(\mathbf{z}, \mu^{\Omega_{k^\star,k',2}}_{[1,2,3,4,5,7]}, \Sigma^{\Omega_{k^\star,k',2}}_{[1,2,3,4,5,7]}\right)\mathbf{dz}$$
$$+ \int_{l_1}^{u_1}\int_{u_2}^{\infty}\int_{u_1}^{\infty}\int_{-\infty}^{0}\int_{l_1}^{u_1}\int_{-\infty}^{0}\int_{u_2}^{\infty} \phi\left(\mathbf{z}, \mu^{\Omega_{k^\star,k',2}}, \Sigma^{\Omega_{k^\star,k',2}}\right)\mathbf{dz},$$

where

$$\mu^{\Omega_{k^\star,k',2}} = \left\{\frac{\sqrt{n}(\mu_{k'}-\mu_0)}{\sigma\sqrt{2}}, \frac{\sqrt{n}(\mu_{k'}-\mu_0)}{\sigma}, \frac{\sqrt{n}(\mu_{k^\star}-\mu_0)}{\sigma\sqrt{2}}, \frac{\sqrt{n}(\mu_{k^\star}-\mu_{k'})}{\sigma\sqrt{2}},\right.$$
$$\left.\frac{\sqrt{n}(\mu_{k_1}-\mu_0)}{\sigma\sqrt{2}}, \frac{\sqrt{n}(\mu_{k_1}-\mu_{k'})}{\sigma}, \frac{\sqrt{n}(\mu_{k^\star}-\mu_{k'})}{\sigma}\right\},$$

and

$$\Sigma^{\Omega_{k^\star,k',2}} = \begin{pmatrix} 1 & \sqrt{\frac{1}{2}} & 0 & 0 & \frac{1}{2} & -\frac{1}{2}\sqrt{\frac{1}{2}} & 0 \\ \sqrt{\frac{1}{2}} & 1 & \frac{1}{2}\sqrt{\frac{1}{2}} & -\frac{1}{2}\sqrt{\frac{1}{2}} & \frac{1}{2}\sqrt{\frac{1}{2}} & -\frac{1}{2} & -\frac{1}{4} \\ 0 & \frac{1}{2}\sqrt{\frac{1}{2}} & 1 & \frac{1}{2} & 0 & 0 & \frac{1}{2}\sqrt{\frac{1}{2}} \\ 0 & -\frac{1}{2}\sqrt{\frac{1}{2}} & \frac{1}{2} & 1 & 0 & \frac{1}{2}\sqrt{\frac{1}{2}} & \sqrt{\frac{1}{2}} \\ \frac{1}{2} & \frac{1}{2}\sqrt{\frac{1}{2}} & 0 & 0 & 1 & \frac{1}{2}\sqrt{\frac{1}{2}} & 0 \\ -\frac{1}{2}\sqrt{\frac{1}{2}} & -\frac{1}{2} & 0 & \frac{1}{2}\sqrt{\frac{1}{2}} & \frac{1}{2}\sqrt{\frac{1}{2}} & 1 & \frac{1}{4} \\ 0 & -\frac{1}{4} & \frac{1}{2}\sqrt{\frac{1}{2}} & \sqrt{\frac{1}{2}} & 0 & \frac{1}{4} & 1 \end{pmatrix}.$$

The conditional power when only using new data is

$$P(E^{\star 4}_{k^\star,k',1}) = \int_{u_2}^{\infty} \phi\left\{\mathbf{z}, \frac{\sqrt{n}(\mu_{k^\star}-\mu_{k'})}{\sigma\sqrt{2}}, 1\right\}\mathbf{dz}.$$

Web Appendix L.4.3 Overall power

When studying the overall power if the treatment with the greatest effect starts at the beginning of the trial we define, k_1 be the other treatment that starts the trial at the beginning, and let k_2 be the treatment which starts after the first stage. Then the probability treatment $k^\star$ becomes the new control at the first stage is

$$\Xi_{k,1} = \int_{u_1}^{\infty}\int_{-\infty}^{0} \phi\Big(\mathbf{z}, \mu^{\Xi_{k,1}}, \Sigma^{\Xi_{k,1}}\Big)\mathbf{dz},$$

where

$$\Sigma^{\Xi_{k,1}} = \begin{pmatrix} 1 & -\frac{1}{2} \\ -\frac{1}{2} & 1 \end{pmatrix}.$$

and

$$\mu^{\Xi_{k,1}} = \left\{ \frac{\sqrt{n}(\mu_{k^\star} - \mu_0)}{\sigma\sqrt{2}}, \frac{\sqrt{n}(\mu_{k_1} - \mu_{k^\star})}{\sigma\sqrt{2}} \right\}.$$

The probability treatment $k^\star$ becomes the new control at the second stage is

$$\begin{aligned}
\Xi_{k,2} =& \int_{l_1}^{u_1}\int_{u_2}^{\infty}\int_{-\infty}^{l_1}\int_{-\infty}^{u_1} \phi\Big(\mathbf{z}, \mu^{\Xi_{k,2}}_{[1,2,3,5]}, \Sigma^{\Xi_{k,2}}_{[1,2,3,5]}\Big)\mathbf{dz} \\
&+ \int_{l_1}^{u_1}\int_{u_2}^{\infty}\int_{l_1}^{u_1}\int_{-\infty}^{0}\int_{-\infty}^{u_1} \phi\Big(\mathbf{z}, \mu^{\Xi_{k,2}}_{[1,2,3,4,5]}, \Sigma^{\Xi_{k,2}}_{[1,2,3,4,5]}\Big)\mathbf{dz} \\
&+ \int_{l_1}^{u_1}\int_{u_2}^{\infty}\int_{-\infty}^{l_1}\int_{u_1}^{\infty}\int_{-\infty}^{0} \phi\Big(\mathbf{z}, \mu^{\Xi_{k,2}}_{[1,2,3,5,6]}, \Sigma^{\Xi_{k,2}}_{[1,2,3,5,6]}\Big)\mathbf{dz} \\
&+ \int_{l_1}^{u_1}\int_{u_2}^{\infty}\int_{l_1}^{u_1}\int_{-\infty}^{0}\int_{u_1}^{\infty}\int_{-\infty}^{0} \phi\Big(\mathbf{z}, \mu^{\Xi_{k,2}}, \Sigma^{\Xi_{k,2}}\Big)\mathbf{dz},
\end{aligned}$$

where

$$\Sigma^{\Xi_{k,2}} = \begin{pmatrix}
1 & \sqrt{\frac{1}{2}} & \frac{1}{2} & -\frac{1}{2}\sqrt{\frac{1}{2}} & 0 & 0 \\
\sqrt{\frac{1}{2}} & 1 & \frac{1}{2}\sqrt{\frac{1}{2}} & -\frac{1}{2} & \frac{1}{2}\sqrt{\frac{1}{2}} & -\frac{1}{2}\sqrt{\frac{1}{2}} \\
\frac{1}{2} & \frac{1}{2}\sqrt{\frac{1}{2}} & 1 & \frac{1}{2}\sqrt{\frac{1}{2}} & \frac{1}{2} & 0 \\
-\frac{1}{2}\sqrt{\frac{1}{2}} & -\frac{1}{2} & \frac{1}{2}\sqrt{\frac{1}{2}} & 1 & 0 & \frac{1}{2}\sqrt{\frac{1}{2}} \\
0 & \frac{1}{2}\sqrt{\frac{1}{2}} & 0 & 0 & 1 & \frac{1}{2} \\
0 & \frac{1}{2}\sqrt{\frac{1}{2}} & 0 & \frac{1}{2}\sqrt{\frac{1}{2}} & \frac{1}{2} & 1
\end{pmatrix}.$$

and

$$\begin{aligned}
\mu^{\Xi_{k,2}} = \Bigg\{ & \frac{\sqrt{n}(\mu_{k^\star} - \mu_0)}{\sigma\sqrt{2}}, \frac{\sqrt{n}(\mu_{k^\star} - \mu_0)}{\sigma}, \frac{\sqrt{n}(\mu_{k_1} - \mu_0)}{\sigma\sqrt{2}}, \frac{\sqrt{n}(\mu_{k_1} - \mu_{k^\star})}{\sigma}, \\
& \frac{\sqrt{n}(\mu_{k_2} - \mu_0)}{\sigma\sqrt{2}}, \frac{\sqrt{n}(\mu_{k_2} - \mu_{k^\star})}{\sigma\sqrt{2}} \Bigg\}.
\end{aligned}$$

Therefore the overall power for treatment $k^\star$ given it starts the trial at the start is

$$\sum_{j^\star=1}^{2} \Xi_{k^\star,j^\star} + \Omega_{k^\star,k'=\{1,2\}/k^\star,1}.$$

Now we consider when $k^\star$ is the treatment which is added later (i.e treatment 3). Now k_1 and k_2 are both for the treatments which began the trial. The probability treatment $k^\star$ becomes the new control at its first stage is:

$$\begin{aligned}
\Xi_{k,1} = &\int_{u_1}^{\infty}\int_{-\infty}^{l_1}\int_{-\infty}^{l_1} \phi\Big(\mathbf{z}, \mu_{[1,2,5]}^{\Xi_{k,1}}, \Sigma_{[1,2,5]}^{\Xi_{k,1}}\Big)\mathbf{dz} \\
&+ \int_{u_1}^{\infty}\int_{-\infty}^{l_1}\int_{l_1}^{u_1}\int_{-\infty}^{u_2} \phi\Big(\mathbf{z}, \mu_{[1,2,5,6]}^{\Xi_{k,1}}, \Sigma_{[1,2,5,6]}^{\Xi_{k,1}}\Big)\mathbf{dz} \\
&+ \int_{u_1}^{\infty}\int_{-\infty}^{l_1}\int_{l_1}^{u_1}\int_{u_2}^{\infty}\int_{-\infty}^{0} \phi\Big(\mathbf{z}, \mu_{[1,2,5,6,7]}^{\Xi_{k,1}}, \Sigma_{[1,2,5,6,7]}^{\Xi_{k,1}}\Big)\mathbf{dz} \\
&+ \int_{u_1}^{\infty}\int_{l_1}^{u_1}\int_{-\infty}^{u_2}\int_{-\infty}^{l_1} \phi\Big(\mathbf{z}, \mu_{[1,2,3,5]}^{\Xi_{k,1}}, \Sigma_{[1,2,3,5]}^{\Xi_{k,1}}\Big)\mathbf{dz} \\
&+ \int_{u_1}^{\infty}\int_{l_1}^{u_1}\int_{-\infty}^{u_2}\int_{l_1}^{u_1}\int_{-\infty}^{u_2} \phi\Big(\mathbf{z}, \mu_{[1,2,3,5,6]}^{\Xi_{k,1}}, \Sigma_{[1,2,3,5,6]}^{\Xi_{k,1}}\Big)\mathbf{dz} \\
&+ \int_{u_1}^{\infty}\int_{l_1}^{u_1}\int_{-\infty}^{u_2}\int_{l_1}^{u_1}\int_{u_2}^{\infty}\int_{-\infty}^{0} \phi\Big(\mathbf{z}, \mu_{[1,2,3,5,6,7]}^{\Xi_{k,1}}, \Sigma_{[1,2,3,5,6,7]}^{\Xi_{k,1}}\Big)\mathbf{dz} \\
&+ \int_{u_1}^{\infty}\int_{l_1}^{u_1}\int_{u_2}^{\infty}\int_{-\infty}^{0}\int_{-\infty}^{l_1} \phi\Big(\mathbf{z}, \mu_{[1,2,3,4,5]}^{\Xi_{k,1}}, \Sigma_{[1,2,3,4,5]}^{\Xi_{k,1}}\Big)\mathbf{dz} \\
&+ \int_{u_1}^{\infty}\int_{l_1}^{u_1}\int_{u_2}^{\infty}\int_{-\infty}^{0}\int_{l_1}^{u_1}\int_{-\infty}^{u_2} \phi\Big(\mathbf{z}, \mu_{[1,2,3,4,5,6]}^{\Xi_{k,1}}, \Sigma_{[1,2,3,4,5,6]}^{\Xi_{k,1}}\Big)\mathbf{dz} \\
&+ \int_{u_1}^{\infty}\int_{l_1}^{u_1}\int_{u_2}^{\infty}\int_{-\infty}^{0}\int_{l_1}^{u_1}\int_{u_2}^{\infty}\int_{-\infty}^{0} \phi\Big(\mathbf{z}, \mu^{\Xi_{k,1}}, \Sigma^{\Xi_{k,1}}\Big)\mathbf{dz},
\end{aligned}$$

where

$$\Sigma^{\Xi_{k,1}} = \begin{pmatrix}
1 & 0 & \frac{1}{2}\sqrt{\frac{1}{2}} & -\frac{1}{2} & 0 & \frac{1}{2}\sqrt{\frac{1}{2}} & -\frac{1}{2} \\
0 & 1 & \sqrt{\frac{1}{2}} & 0 & \frac{1}{2} & \frac{1}{2}\sqrt{\frac{1}{2}} & 0 \\
\frac{1}{2}\sqrt{\frac{1}{2}} & \sqrt{\frac{1}{2}} & 1 & \frac{1}{2}\sqrt{\frac{1}{2}} & \frac{1}{2}\sqrt{\frac{1}{2}} & \frac{1}{2} & 0 \\
-\frac{1}{2} & 0 & \frac{1}{2}\sqrt{\frac{1}{2}} & 1 & 0 & 0 & \frac{1}{2} \\
0 & \frac{1}{2} & \frac{1}{2}\sqrt{\frac{1}{2}} & 0 & 1 & \sqrt{\frac{1}{2}} & 0 \\
\frac{1}{2}\sqrt{\frac{1}{2}} & \frac{1}{2}\sqrt{\frac{1}{2}} & \frac{1}{2} & 0 & \sqrt{\frac{1}{2}} & 1 & \frac{1}{2}\sqrt{\frac{1}{2}} \\
-\frac{1}{2} & 0 & 0 & \frac{1}{2} & 0 & \frac{1}{2}\sqrt{\frac{1}{2}} & 1
\end{pmatrix},$$

and

$$\mu^{\Xi_{k,1}} = \Bigg\{ \frac{\sqrt{n}(\mu_{k^\star} - \mu_0)}{\sigma\sqrt{2}}, \frac{\sqrt{n}(\mu_{k_1} - \mu_0)}{\sigma\sqrt{2}}, \frac{\sqrt{n}(\mu_{k_1} - \mu_0)}{\sigma}, \frac{\sqrt{n}(\mu_{k_1} - \mu_{k^\star})}{\sigma\sqrt{2}}, \frac{\sqrt{n}(\mu_{k_2} - \mu_0)}{\sigma\sqrt{2}}, \frac{\sqrt{n}(\mu_{k_2} - \mu_0)}{\sigma}, \frac{\sqrt{n}(\mu_{k_2} - \mu_{k^\star})}{\sigma\sqrt{2}} \Bigg\}.$$

The probability treatment $k^\star$ becomes the new control at its second stage is:

$$\begin{aligned}\Xi_{k,2} =& \int_{l_1}^{u_1}\int_{u_2}^{\infty}\int_{-\infty}^{l_1}\int_{-\infty}^{l_1} \phi\left(\mathbf{z}, \mu^{\Xi_{k,2}}_{[1,2,3,5]}, \Sigma^{\Xi_{k,2}}_{[1,2,3,5]}\right)\mathbf{dz}\\ &+ \int_{l_1}^{u_1}\int_{u_2}^{\infty}\int_{-\infty}^{l_1}\int_{l_1}^{u_1}\int_{-\infty}^{u_2} \phi\left(\mathbf{z}, \mu^{\Xi_{k,2}}_{[1,2,3,5,6]}, \Sigma^{\Xi_{k,2}}_{[1,2,3,5,6]}\right)\mathbf{dz}\\ &+ \int_{l_1}^{u_1}\int_{u_2}^{\infty}\int_{l_1}^{u_1}\int_{-\infty}^{u_2}\int_{-\infty}^{l_1} \phi\left(\mathbf{z}, \mu^{\Xi_{k,2}}_{[1,2,3,4,5]}, \Sigma^{\Xi_{k,2}}_{[1,2,3,4,5]}\right)\mathbf{dz}\\ &+ \int_{l_1}^{u_1}\int_{u_2}^{\infty}\int_{l_1}^{u_1}\int_{-\infty}^{u_2}\int_{l_1}^{u_1}\int_{-\infty}^{u_2} \phi\left(\mathbf{z}, \mu^{\Xi_{k,2}}, \Sigma^{\Xi_{k,2}}\right)\mathbf{dz},\end{aligned}$$

where

$$\Sigma^{\Xi_{k,2}} = \begin{pmatrix} 1 & \sqrt{\frac{1}{2}} & 0 & \frac{1}{2}\sqrt{\frac{1}{2}} & 0 & \frac{1}{2}\sqrt{\frac{1}{2}} \\ \sqrt{\frac{1}{2}} & 1 & 0 & \frac{1}{4} & 0 & \frac{1}{4} \\ 0 & 0 & 1 & \sqrt{\frac{1}{2}} & \frac{1}{2} & \frac{1}{2}\sqrt{\frac{1}{2}} \\ \frac{1}{2}\sqrt{\frac{1}{2}} & \frac{1}{4} & \sqrt{\frac{1}{2}} & 1 & \frac{1}{2}\sqrt{\frac{1}{2}} & \frac{1}{2} \\ 0 & 0 & \frac{1}{2} & \frac{1}{2}\sqrt{\frac{1}{2}} & 1 & \sqrt{\frac{1}{2}} \\ \frac{1}{2}\sqrt{\frac{1}{2}} & \frac{1}{4} & \frac{1}{2}\sqrt{\frac{1}{2}} & \frac{1}{2} & \sqrt{\frac{1}{2}} & 1 \end{pmatrix},$$

and

$$\begin{aligned}\mu^{\Xi_{k,2}} = \Bigg\{ & \frac{\sqrt{n}(\mu_{k^\star}-\mu_0)}{\sigma\sqrt{2}}, \frac{\sqrt{n}(\mu_{k^\star}-\mu_0)}{\sigma}, \frac{\sqrt{n}(\mu_{k_1}-\mu_0)}{\sigma\sqrt{2}}, \frac{\sqrt{n}(\mu_{k_1}-\mu_0)}{\sigma},\\ & \frac{\sqrt{n}(\mu_{k_2}-\mu_0)}{\sigma\sqrt{2}}, \frac{\sqrt{n}(\mu_{k_2}-\mu_0)}{\sigma}\Bigg\}.\end{aligned}$$

Therefore, the overall power for treatment $k^\star$ given it is the treatment which is added later is

$$\sum_{j^\star=1}^{2} \Xi_{k^\star,j^\star} + \sum_{k'\in\{1,2\}}\sum_{j'=1}^{2} \Omega_{k^\star,k',j'}.$$

Web Appendix L.5 Results

In the results given below it can be seen that for this example there is no benefit in keeping the historic data for both overall and conditional power.

Web Appendix L.5.1 Conditional power for treatment 2 against treatment 1 after the first stage

The results of using different values of $\mu_3-\mu_0$ are studied. The values studied are $\mu_3-\mu_0=-\theta_0$, $\mu_3-\mu_0=0$ and $\mu_3-\mu_0=\theta_0$. The conditional power for treatment 2 given treatment 1 has become the new control at stage 1 when using all the data is given in Figure 13. The conditional power for treatment 2 given treatment 1 has become the new control at stage 1 when using only the new data is given in Figure 14. The difference in conditional power for treatment 2 given treatment 1 has become the new control at stage 1 is given in Figure 15.

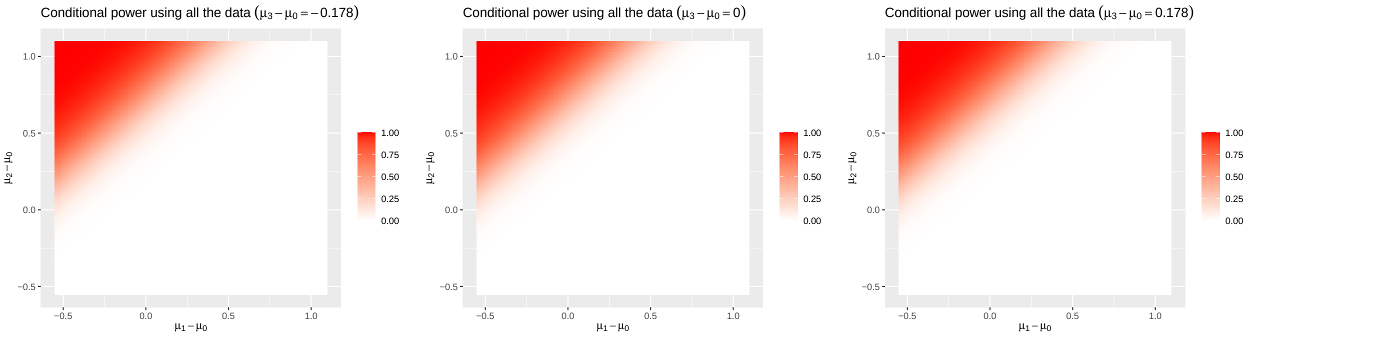

Web Figure 13. For multiple values of $\mu_3 - \mu_0$ with treatment 3 added later: the conditional power for treatment 2 given that treatment 1 has gone forward at the first stage when all the data is retained.

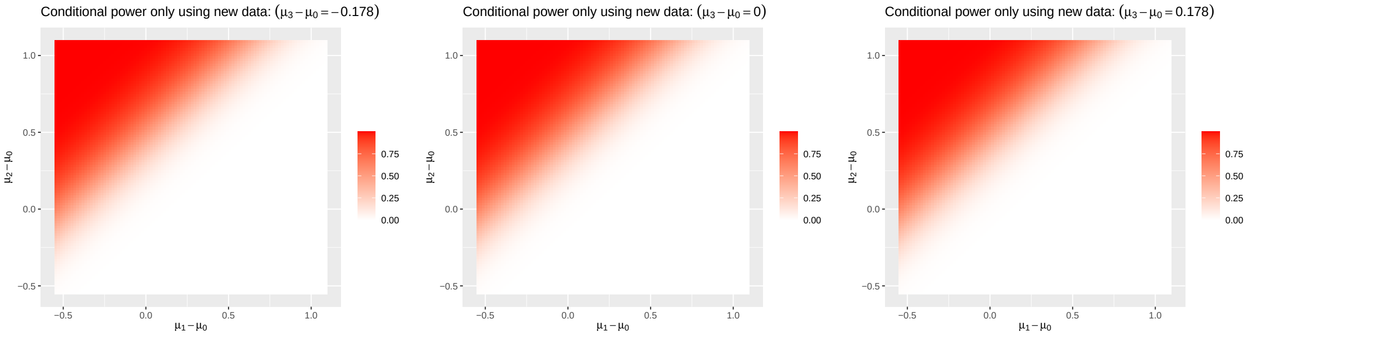

Web Figure 14. For multiple values of $\mu_3 - \mu_0$ with treatment 3 added later: the conditional power for treatment 2 given that treatment 1 has gone forward at the first stage when only the data post the change in control is used.

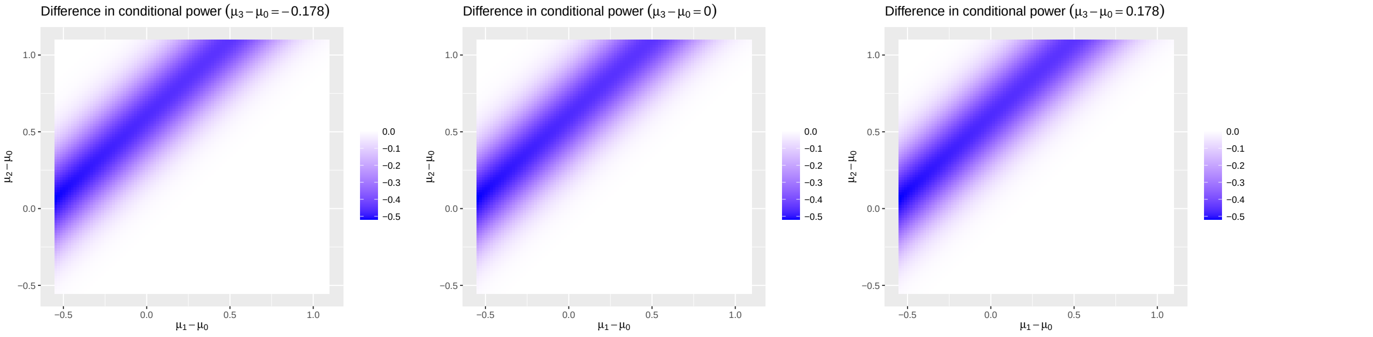

Web Figure 15. For multiple values of $\mu_3 - \mu_0$ with treatment 3 added later: the difference in conditional power between keeping the data pre change and not.

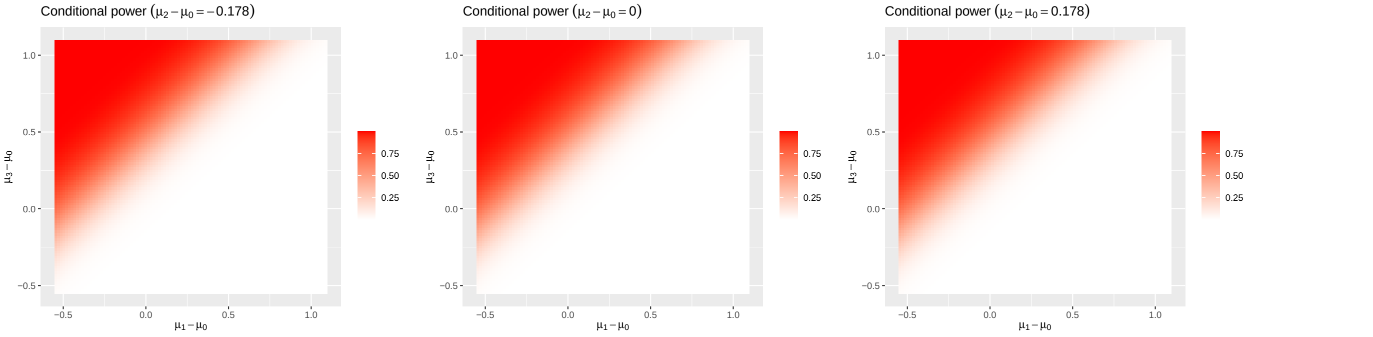

Web Figure 16. For multiple values of $\mu_2 - \mu_0$ with treatment 3 added later: the conditional power for treatment 3 given that treatment 1 has gone forward at the first stage.

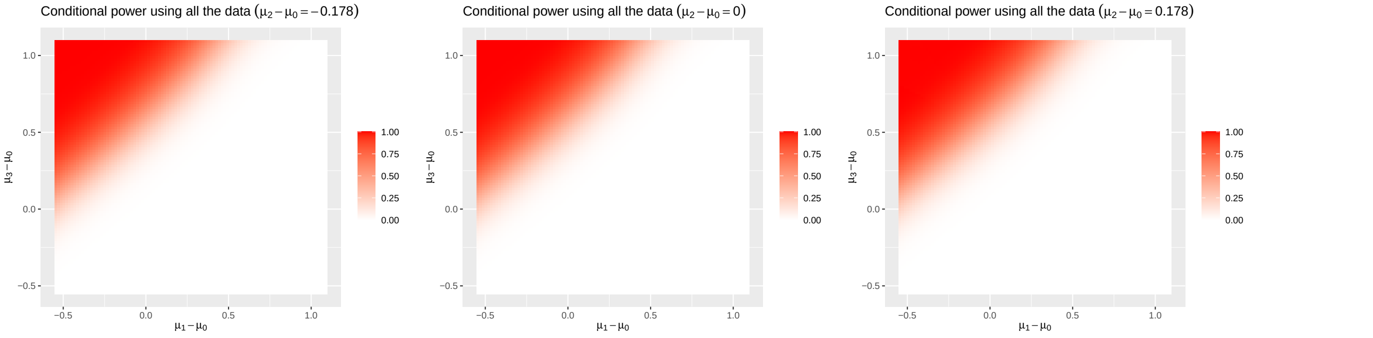

Web Figure 17. For multiple values of $\mu_2 - \mu_0$ with treatment 3 added later: the conditional power for treatment 3 given that treatment 1 has gone forward at the second stage when all the data is retained.

Web Appendix L.5.2 Conditional power for treatment 3 against treatment 1 after the first stage

The results of using different values of $\mu_2 - \mu_0$ are studied. The values studied are $\mu_2 - \mu_0 = -\theta_0$, $\mu_2 - \mu_0 = 0$ and $\mu_2 - \mu_0 = \theta_0$. The conditional power for treatment 3 given treatment 1 has become the new control at stage 1 is given in Figure 16.

Web Appendix L.5.3 Conditional power for treatment 3 against treatment 1 after the second stage

The results of using different values of $\mu_2 - \mu_0$ are studied. The values studied are $\mu_2 - \mu_0 = -\theta_0$, $\mu_2 - \mu_0 = 0$ and $\mu_2 - \mu_0 = \theta_0$. The conditional power for treatment 3 given treatment 1 has become the new control at stage 2 when using all the data is given in Figure 17. The conditional power for treatment 3 given treatment 1 has become the new control at stage 1 when using only the new data is given in Figure 18. The difference in conditional power for treatment 3 given treatment 1 has become the new control at stage 1 is given in Figure 19.

Web Appendix L.5.4 Overall power for treatment 2 against treatment 1

The results of using different values of $\mu_3 - \mu_0$ are studied. The values studied are $\mu_3 - \mu_0 = -\theta_0$, $\mu_3 - \mu_0 = 0$ and $\mu_3 - \mu_0 = \theta_0$. The overall power when using all the data is given in Figure

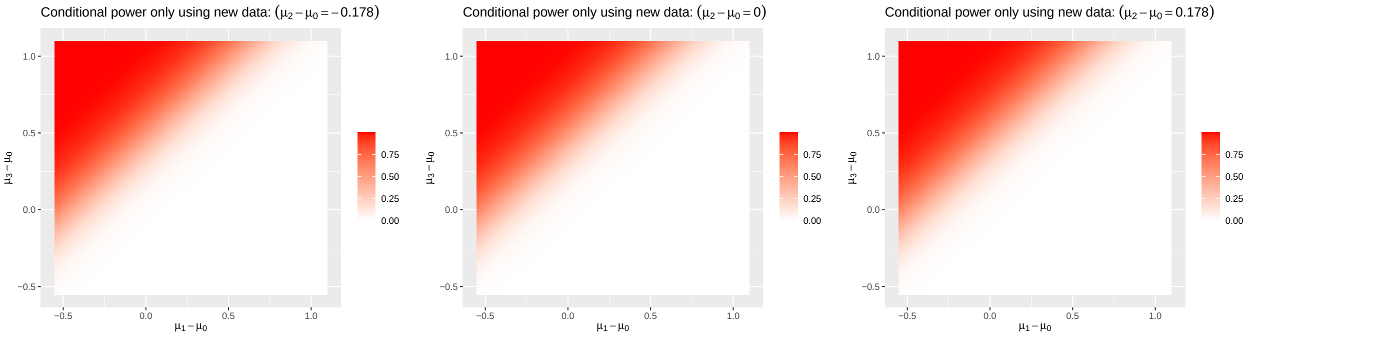

Web Figure 18. For multiple values of $\mu_2 - \mu_0$ with treatment 3 added later: the conditional power for treatment 3 given that treatment 1 has gone forward at the second stage when only the data post the change in control is used.

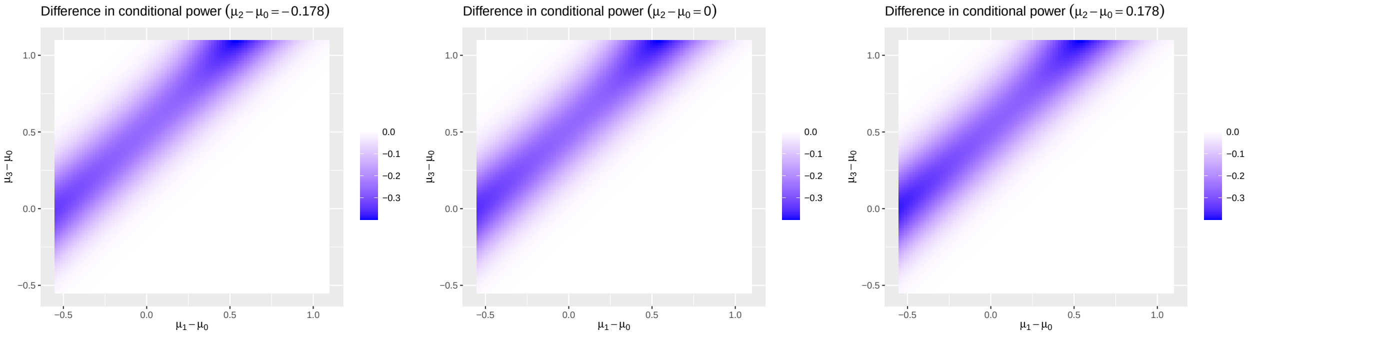

Web Figure 19. For multiple values of $\mu_2 - \mu_0$ with treatment 3 added later: the difference in conditional power between keeping the data pre change and not.

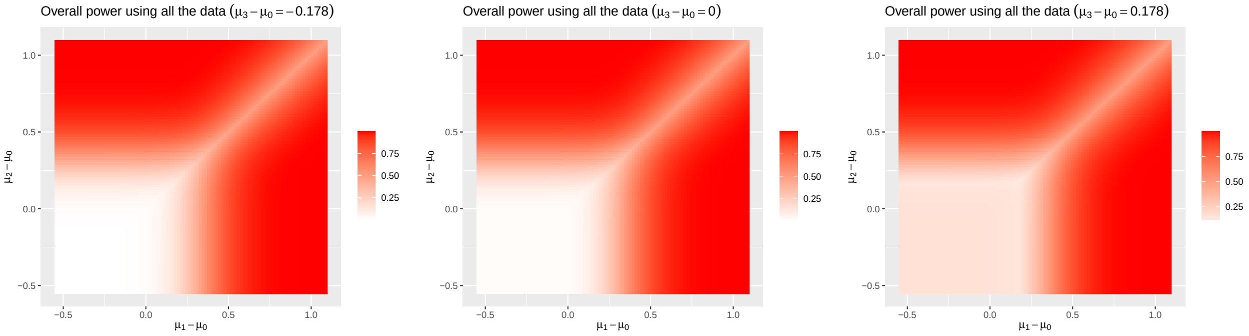

Web Figure 20. For multiple values of $\mu_3 - \mu_0$ with treatment 3 added later: the overall power when all the data is retained.

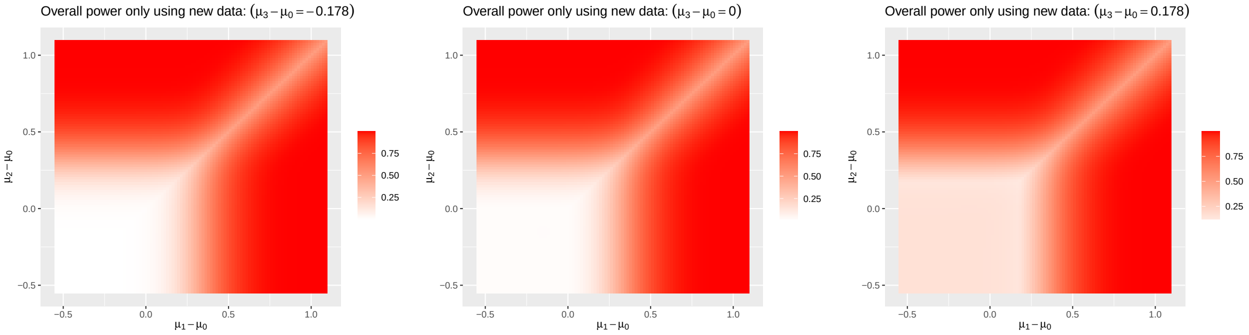

Web Figure 21. For multiple values of $\mu_3 - \mu_0$ with treatment 3 added later: the overall power when only the data post the change in control is used.

20. The overall power when using only the new data is given in Figure 21. The difference in overall power is given in Figure 22.

Web Appendix L.5.5 Overall power for treatment 3 against treatment 1

The results of using different values of $\mu_2 - \mu_0$ are studied. The values studied are $\mu_2 - \mu_0 = -\theta_0$, $\mu_2 - \mu_0 = 0$ and $\mu_2 - \mu_0 = \theta_0$. The overall power when using all the data is given in Figure 23. The overall power when using only the new data is given in Figure 24. The difference in overall power is given in Figure 25.

Web Appendix L.6 Added later with no interim analyses

In this example each treatment only has one analysis as is illustrated in Figure 26. Therefore when treatment 1 and 2 have their analysis treatment 3 is halfway through recruitment and is not studied at this point. The upper and lower boundaries are found using the method in Greenstreet et al. (2024) to once again control the FWER at 5%. They are $u_1 = l_1 = 2.089$. In addition each active treatment gets 78 patients so the maximum sample size is 351 in order to control the pairwise power at 90% (Greenstreet et al., 2025).

For the conditional power there is only one point where this is non zero and this is if either treatment 1 or 2 go forward at their final analysis with treatment 3 being the one of interest. If

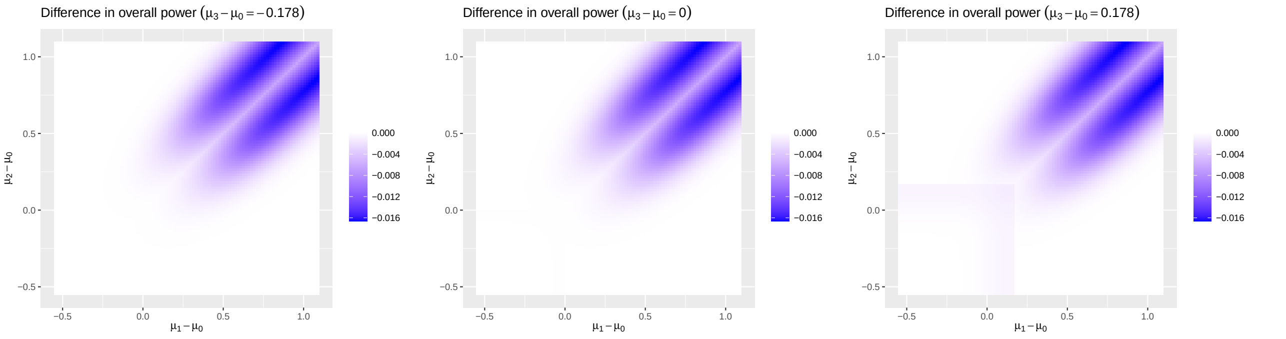

Web Figure 22. For multiple values of $\mu_3 - \mu_0$ with treatment 3 added later: the difference in overall power between keeping the data pre change and not.

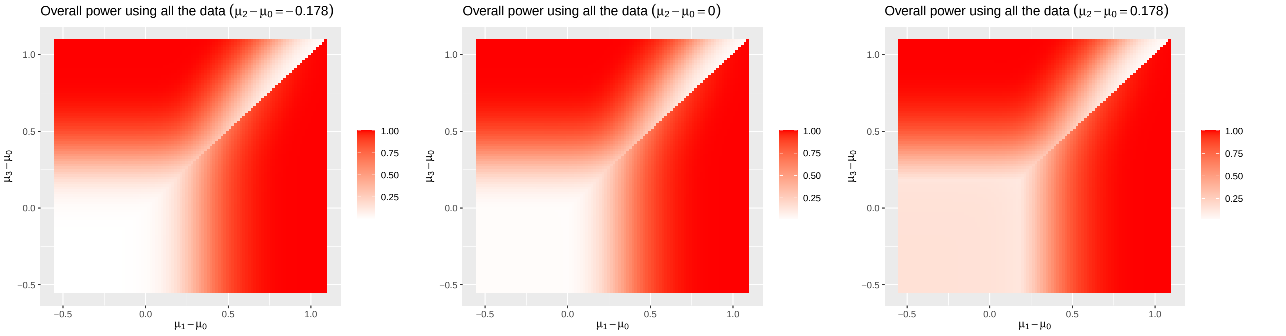

Web Figure 23. For multiple values of $\mu_2 - \mu_0$ with treatment 3 added later: the overall power when all the data is retained.

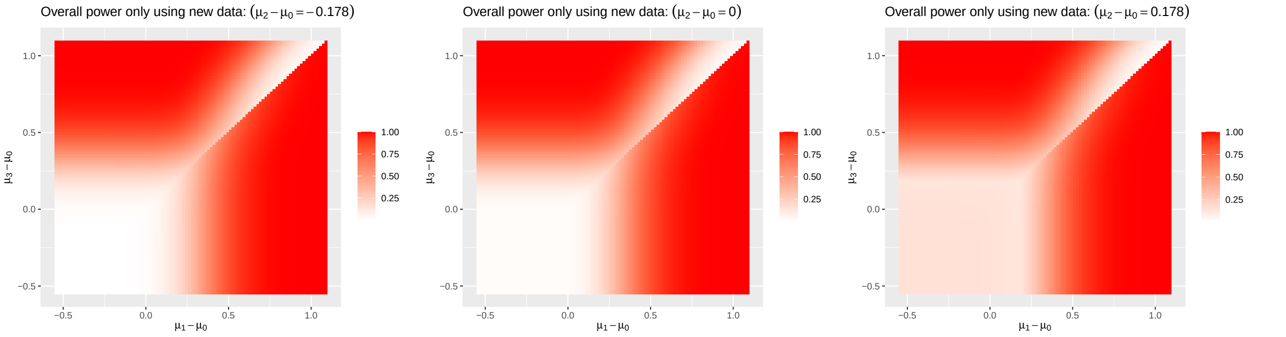

Web Figure 24. For multiple values of $\mu_2 - \mu_0$ with treatment 3 added later: the overall power when only the data post the change in control is used.

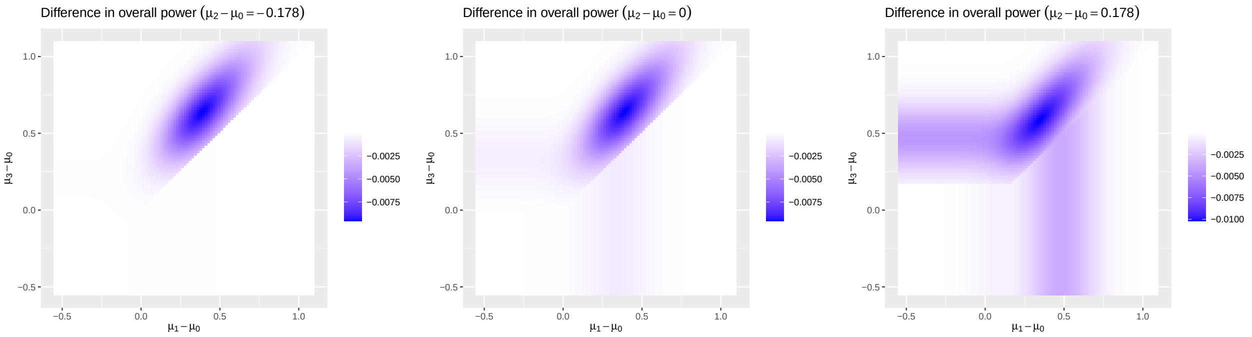

Web Figure 25. For multiple values of $\mu_2 - \mu_0$ with treatment 3 added later: the difference in overall power between keeping the data pre change and not.

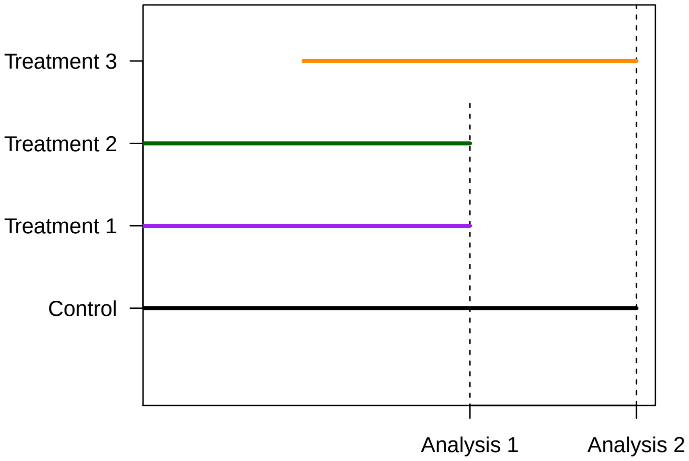

Web Figure 26. Illustration of the motivating trial when one treatment starts after the first stage and all the treatments only have one analysis.

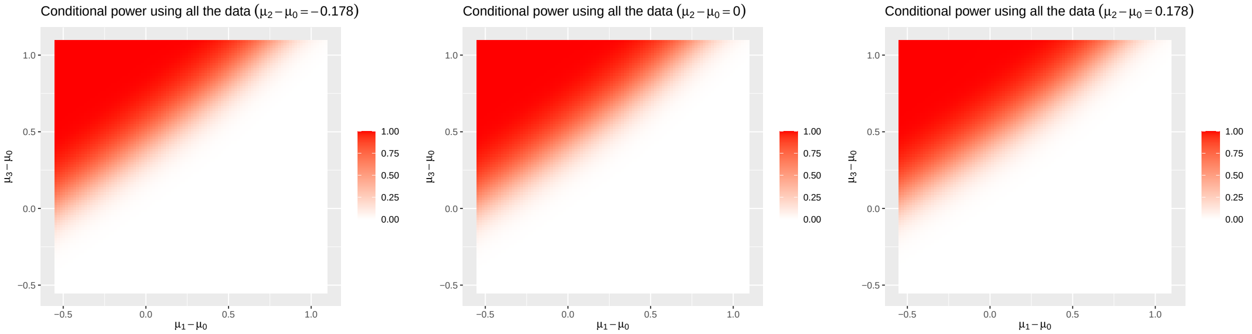

Web Figure 27. For multiple values of $\mu_2-\mu_0$ with treatment 3 added later and each treatment only has 1 analysis: the conditional power for treatment 3 given that treatment 1 has gone forward when all the data is retained.

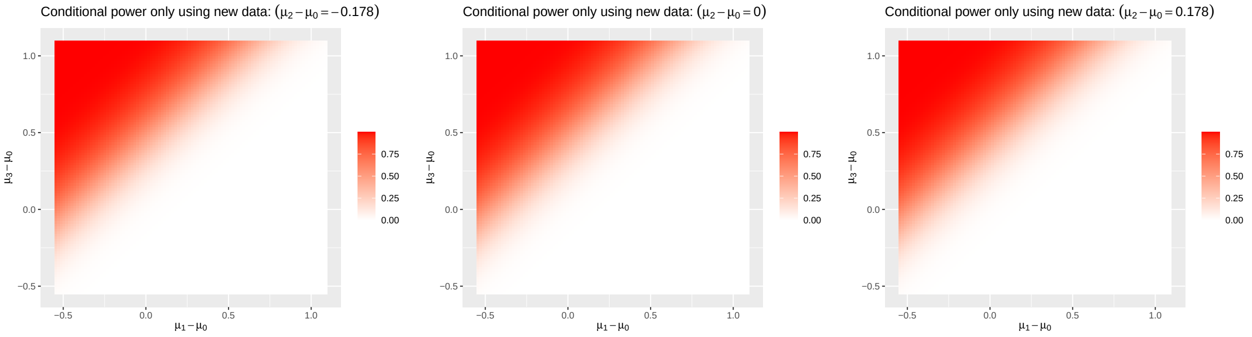

Web Figure 28. For multiple values of $\mu_2-\mu_0$ with treatment 3 added later and each treatment only has 1 analysis: the conditional power for treatment 3 given that treatment 1 has gone forward when only the data post the change in control is used.

one assumes it was treatment 1 that goes forward the conditional power for treatment 3 if all the data is used, for multiple values of $\mu_2 - \mu_0$, can be seen in Figure 27. Similarly in Figure 28 the conditional power if only the data post change is used. Figure 29 shows the difference in conditional power. As can be seen in Figure 29 now retaining the information can have a positive effect on the conditional power of the trial.

In Figure 30 overall power when comparing treatment 1 and 2 is presented for multiple values of $\mu_3 - \mu_0$. The only difference between the overall power when retaining all the data is from treatment 3. Therefore only one set of results when comparing treatment 1 and 2 are presented. However in Figure 31 and Figure 32 the overall power when keeping all the data or only keeping the new data are shown comparing treatment 1 and 3, respectively. In Figure 33 the difference in overall power is shown. This shows in this example there is almost always advantage to keeping the historic data with a power increase of potentially more then 2.5%.

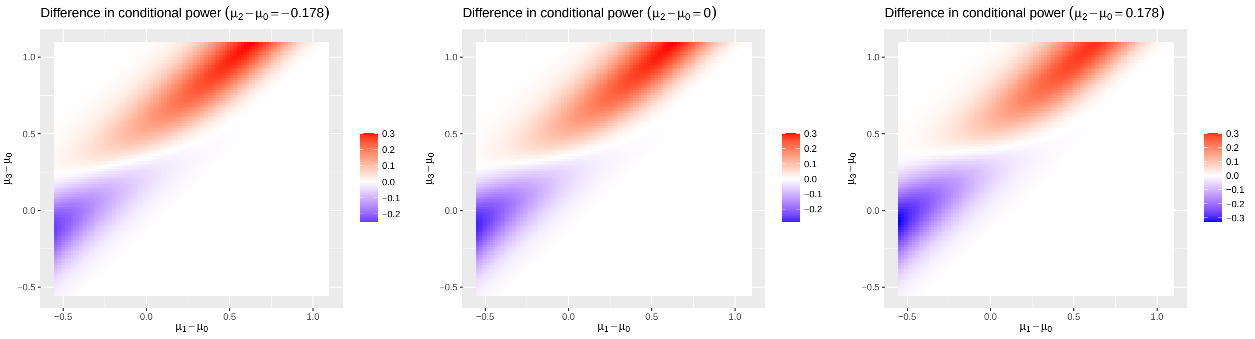

Web Figure 29. For multiple values of $\mu_2 - \mu_0$ with treatment 3 added later and each treatment only has 1 analysis: the difference in conditional power between keeping the data pre change and not.

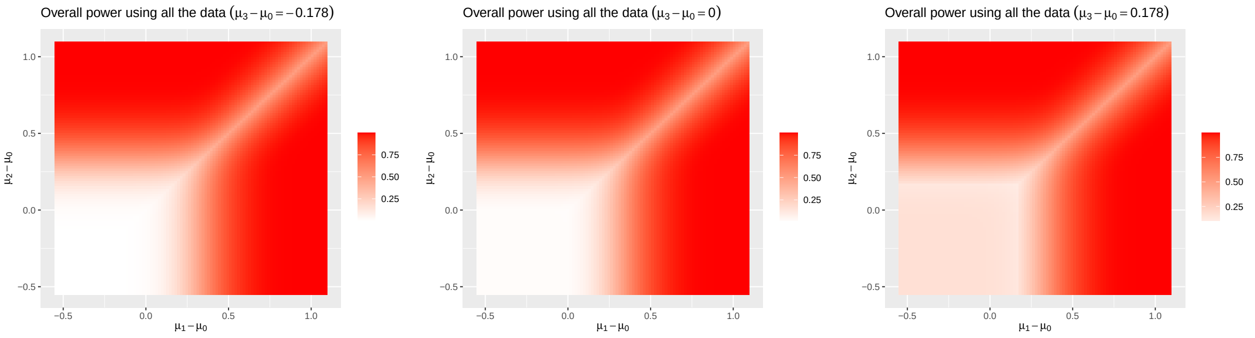

Web Figure 30. For multiple values of $\mu_3 - \mu_0$ with treatment 3 added later and each treatment only has 1 analysis: the overall power when only the data post the change in control is used.

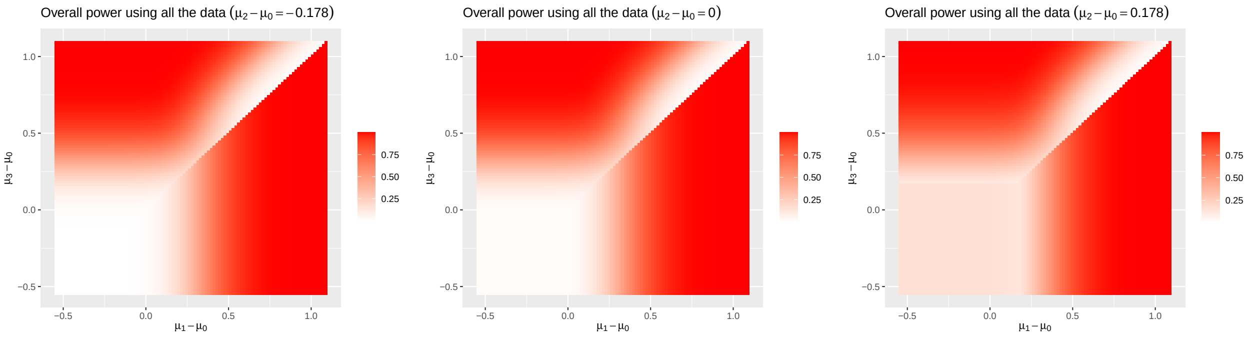

Web Figure 31. For multiple values of $\mu_2 - \mu_0$ with treatment 3 added later and each treatment only has 1 analysis: the overall power when all the data is retained.

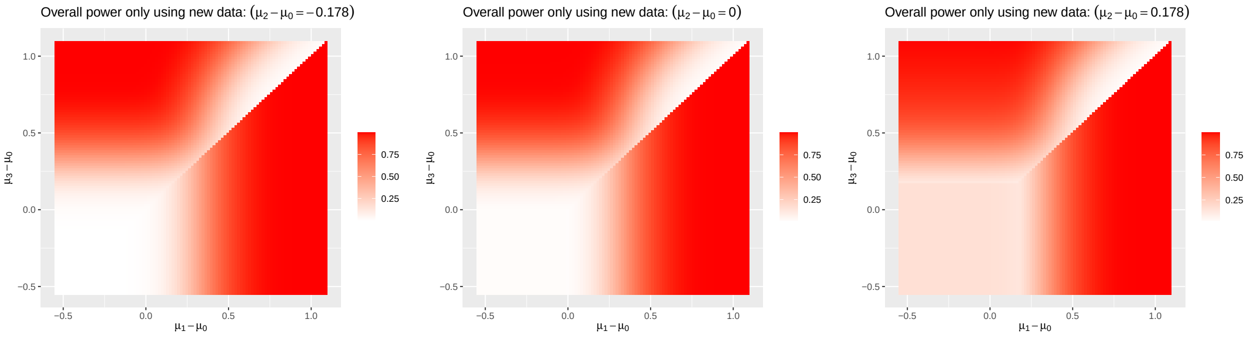

Web Figure 32. For multiple values of $\mu_2 - \mu_0$ with treatment 3 added later and each treatment only has 1 analysis: the overall power when only the data post the change in control is used.

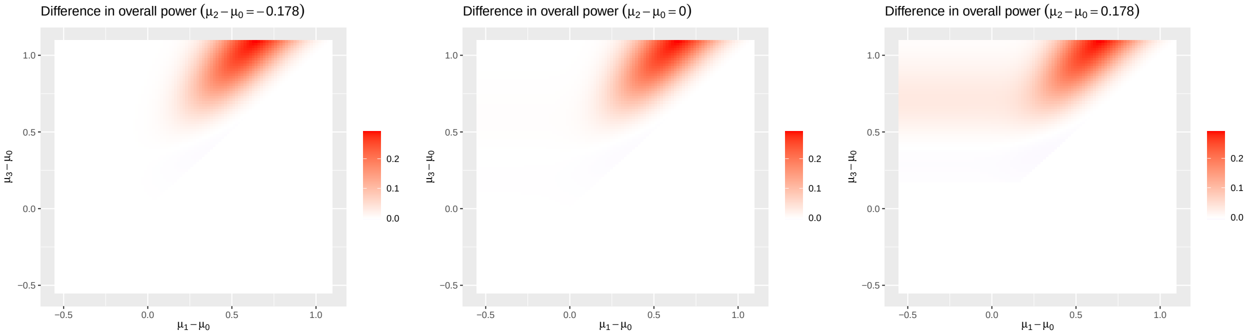

Web Figure 33. For multiple values of $\mu_2 - \mu_0$ with treatment 3 added later and each treatment only has 1 analysis: the difference in overall power between keeping the data pre change and not.

References

Greenstreet, P., Jaki, T., Bedding, A., Harbron, C., and Mozgunov, P. (2024). A multi-arm multi-stage platform design that allows preplanned addition of arms while still controlling the family-wise error. *Statistics in Medicine*, 43(19):3613–3632.

Greenstreet, P., Jaki, T., Bedding, A., and Mozgunov, P. (2025). A Preplanned Multi-Stage Platform Trial for Discovering Multiple Superior Treatments With Control of FWER and Power. *Biometrical Journal*, 67(1):e70025.

Magirr, D., Jaki, T., and Whitehead, J. (2012). A generalized Dunnett test for multi-arm multi-stage clinical studies with treatment selection. *Biometrika*, 99(2):494–501.

O'Brien, P. C. and Fleming, T. R. (1979). A Multiple Testing Procedure for Clinical Trials. *Biometrics*, 35(3):549–556.

Pushpakom, S., Kolamunnage-Dona, R., Taylor, C., Foster, T., Spowart, C., García-Fiñana, M., et al. (2020). TAILoR (TelmisArtan and InsuLin Resistance in Human Immunodeficiency Virus [HIV]): An Adaptive-design, Dose-ranging Phase IIb Randomized Trial of Telmisartan for the Reduction of Insulin Resistance in HIV-positive Individuals on Combination Antiretroviral Therapy. *Clinical infectious diseases*, 70(10):2062–2072.

Pushpakom, S. P., Taylor, C., Kolamunnage-Dona, R., Spowart, C., Vora, J., García-Fiñana, M., et al. (2015). Telmisartan and Insulin Resistance in HIV (TAILoR): protocol for a dose-ranging phase II randomised open-labelled trial of telmisartan as a strategy for the reduction of insulin resistance in HIV-positive individuals on combination antiretroviral therapy. *BMJ open*, 5(10):e009566.

Stallard, N. and Todd, S. (2003). Sequential designs for phase III clinical trials incorporating treatment selection. *Statistics in medicine*, 22(5):689–703.

Wason, J. M. S. and Jaki, T. (2012). Optimal design of multi-arm multi-stage trials. *Statistics in Medicine*, 31(30):4269–4279.

Whitehead, J. (1997). The Design and Analysis of Sequential Clinical Trials. *Biometrics*, 53(4):1564.
